# Enhancing the Backbone Coplanarity of n-Type Copolymers for Higher Electron Mobility and Stability in Organic Electrochemical Transistors

**DOI:** 10.1021/acs.chemmater.2c01552

**Published:** 2022-09-27

**Authors:** Iuliana P. Maria, Sophie Griggs, Reem B. Rashid, Bryan D. Paulsen, Jokubas Surgailis, Karl Thorley, Vianna N. Le, George T. Harrison, Craig Combe, Rawad Hallani, Alexander Giovannitti, Alexandra F. Paterson, Sahika Inal, Jonathan Rivnay, Iain McCulloch

**Affiliations:** † Department of Chemistry and Centre for Plastic Electronics, Imperial College London, London SW7 2AZ, U.K.; ‡ Department of Chemistry, Chemistry Research Laboratory, University of Oxford, Oxford OX1 3TA, U.K.; § Department of Biomedical Engineering, Northwestern University, Evanston, Illinois 60208-0001, United States; ∥ Biological and Environmental Science and Engineering Division, King Abdullah University of Science and Technology, Thuwal 23955-6900, Saudi Arabia; ⊥ Department of Chemistry, University of Kentucky, Lexington, Kentucky 40506-0055, United States; # Department of Chemical and Materials Engineering, University of Kentucky, Lexington, Kentucky 40506-0055, United States; ∇ KAUST Solar Center, King Abdullah University of Science and Technology, Thuwal 23955-6900, Saudi Arabia; ○ Department of Materials Science and Engineering, Stanford University, Stanford, California 94305, United States; ◆ Simpson Querrey Institute, Northwestern University, Evanston, Illinois 60611, United States

## Abstract

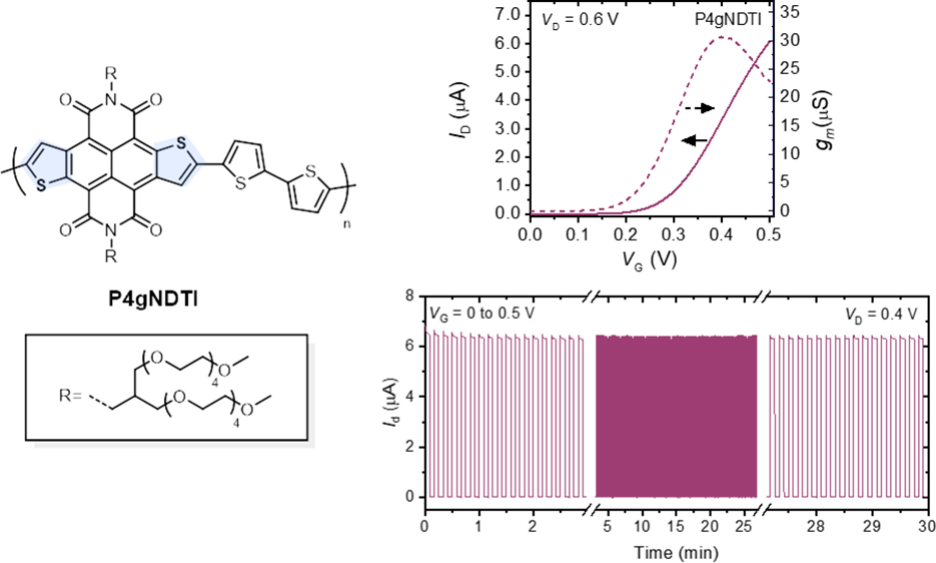

Electron-transporting (n-type) conjugated polymers have recently been applied in numerous electrochemical applications, where both ion and electron transport are required. Despite continuous efforts to improve their performance and stability, n-type conjugated polymers with mixed conduction still lag behind their hole-transporting (p-type) counterparts, limiting the functions of electrochemical devices. In this work, we investigate the effect of enhanced backbone coplanarity on the electrochemical activity and mixed ionic-electronic conduction properties of n-type polymers during operation in aqueous media. Through substitution of the widely employed electron-deficient naphthalene diimide (NDI) unit for the core-extended naphthodithiophene diimide (NDTI) units, the resulting polymer shows a more planar backbone with closer packing, leading to an increase in the electron mobility in organic electrochemical transistors (OECTs) by more than two orders of magnitude. The NDTI-based polymer shows a deep-lying lowest unoccupied molecular orbital level, enabling operation of the OECT closer to 0 V vs Ag/AgCl, where fewer parasitic reactions with molecular oxygen occur. Enhancing the backbone coplanarity also leads to a lower affinity toward water uptake during cycling, resulting in improved stability during continuous electrochemical charging and ON–OFF switching relative to the NDI derivative. Furthermore, the NDTI-based polymer also demonstrates near-perfect shelf-life stability over a month-long test, exhibiting a negligible decrease in both the maximum on-current and transconductance. Our results highlight the importance of polymer backbone design for developing stable, high-performing n-type materials with mixed ionic-electronic conduction in aqueous media.

## Introduction

Recent progress in the development of organic mixed ionic-electronic conductors (OMIECs) continues to drive forward numerous bioelectronic,^1−5^ neuromorphic computing^6,7^ and energy storage technologies.^8,9^ The interest in conjugated polymers for these applications stems from their ability to transport and couple ionic and electronic charge,^10^ as well as their versatility in structural modification. Through chemical design, conjugated polymers can be tailored to support volumetric coupling of electrolyte ions and electronic charges, a property often leveraged by organic electrochemical transistors (OECTs). During the operation of an OECT, an input voltage at the gate electrode is used to control the injection of ionic and electronic charge carriers into the bulk of the OMIEC and reversibly modulate its doping state. The bulk nature of OECT operation leads to high capabilities of transducing and amplifying low potentials (quantified by the transconductance, *g*_m_ = ∂*I*_D_/∂*V*_G_),^11^ rendering them particularly attractive for operation in aqueous media.

In conjugated polymers, the transport of hydrated ions is facilitated by microstructures with free volume that can accommodate ion intercalation^12,13^ or hydrophilic components such as polyelectrolyte phases^14,15^ and polar side chains.^16−21^ Functionalization with ethylene glycol (EG) side chains, in particular, has led to the development of p-type OMIECs with *g*_m_ higher than 1000 S/cm.^22^ Progress of n-type OMIECs has been considerably slower, despite their high demand for implementation in complementary circuit designs, ,^13^ supercapacitors,^23^ or detection of sensing events that generate electrons.^24,25^ The challenge of developing high-performing n-type conjugated polymers for organic electronics has been traditionally attributed to the instability of the polaronic or bipolaronic states in ambient conditions, where parasitic side reactions with water (hydrogen evolution reaction) and molecular oxygen (oxygen reduction reaction, ORR) can lead to a reduction in the number of mobile electrons available and negatively impact the charge transport properties.^26−29^ In the case of OMIECs, an additional design challenge is the need for polymer microstructures which allow volumetric penetration of ions, without compromising the pathways for electron transport. Many current OMIECs undergo extensive swelling due to uptake of hydrated ions and accompanying water molecules, leading to disruption of the connections between crystalline domains and loss of structural order in aqueous electrolytes.^30,31^

A dominant role among n-type OMIECs has been played by conjugated polymers featuring the naphthalene diimide (NDI) unit as the key acceptor building block due to its ability to undergo reversible electrochemical redox reactions in aqueous electrolytes.^32^ Attempts to optimize their mixed ionic-electronic conduction properties have so far involved engineering of the side chain, with a particular focus on tuning the density of EG side chains and their relative position.^16,33^ For example, introduction of alkyl spacers in between their conjugated backbone and the EG-based side chains is an effective strategy to limit detrimental swelling, improve the operational stability,^34^ and charge carrier mobility of the channel material in OECTs operating in aqueous media.^35^

Beyond side chain modification, engineering of the polymer backbone offers many more opportunities to promote closer interchain electronic coupling, improve long-range order, and therefore increase charge carrier mobility. In this context, a successful strategy has been to incorporate rigid electron-deficient building blocks and minimize the rotational torsion between conjugated repeat units. A conformationally locked n-type polymer based on aryl lactam units fused through double bonds demonstrated high transconductance and an electron mobility on the order of 10^–3^ cm^2^ V^–1^ s^–1^ when operated in OECTs.^36^ Recent work has also shown that rigid frameworks are less vulnerable to detrimental swelling and loss of crystallite interconnectivity during operation.^12^ Strategies that can enhance the backbone coplanarity and rigidity of current n-type OMIECs are therefore promising for achieving higher electron mobilities and *g*_m,_ leading to high-performing OECT technologies.

In this work, we investigate the effect of enhanced backbone coplanarity on the electrochemical activity and OECT characteristics of n-type conjugated polymers operating in aqueous media. The naphthodithiophene diimide (NDTI) unit, a thiophene-annulated derivative of NDI,^37^ was chosen as the acceptor unit due to its highly planar molecular structure compared to the NDI unit. The extended effective π-conjugation conferred by a more coplanar backbone was also expected to increase electron affinity of the polymer, thus limiting parasitic side reactions of the doped polymer with molecular oxygen during device operation in ambient conditions. Using density functional theory (DFT) analysis, we confirmed that NDTI units promote a more coplanar geometry and reduce torsional disorder along the polymer backbone relative to NDI units. Functionalization with branched tetraethylene glycol side chains afforded an NDTI-T2 polymer with deep-lying LUMO energy levels of 4.17 eV and early onset for reversible electrochemical reduction in aqueous electrolytes. Furthermore, increasing the planarity of the backbone led to more controlled swelling and improved stability during extended cycling for the NDTI-based polymer than its NDI analogue in aqueous electrolytes. The NDTI-T2 copolymer also shows an increase in the μC* product and thickness-normalized transconductance by more than two orders of magnitude and excellent long-term stability in OECTs, demonstrating that backbone coplanarity is a key parameter for designing stable, high performing materials for OECTs.

## Results and Discussion

**Scheme 1:**
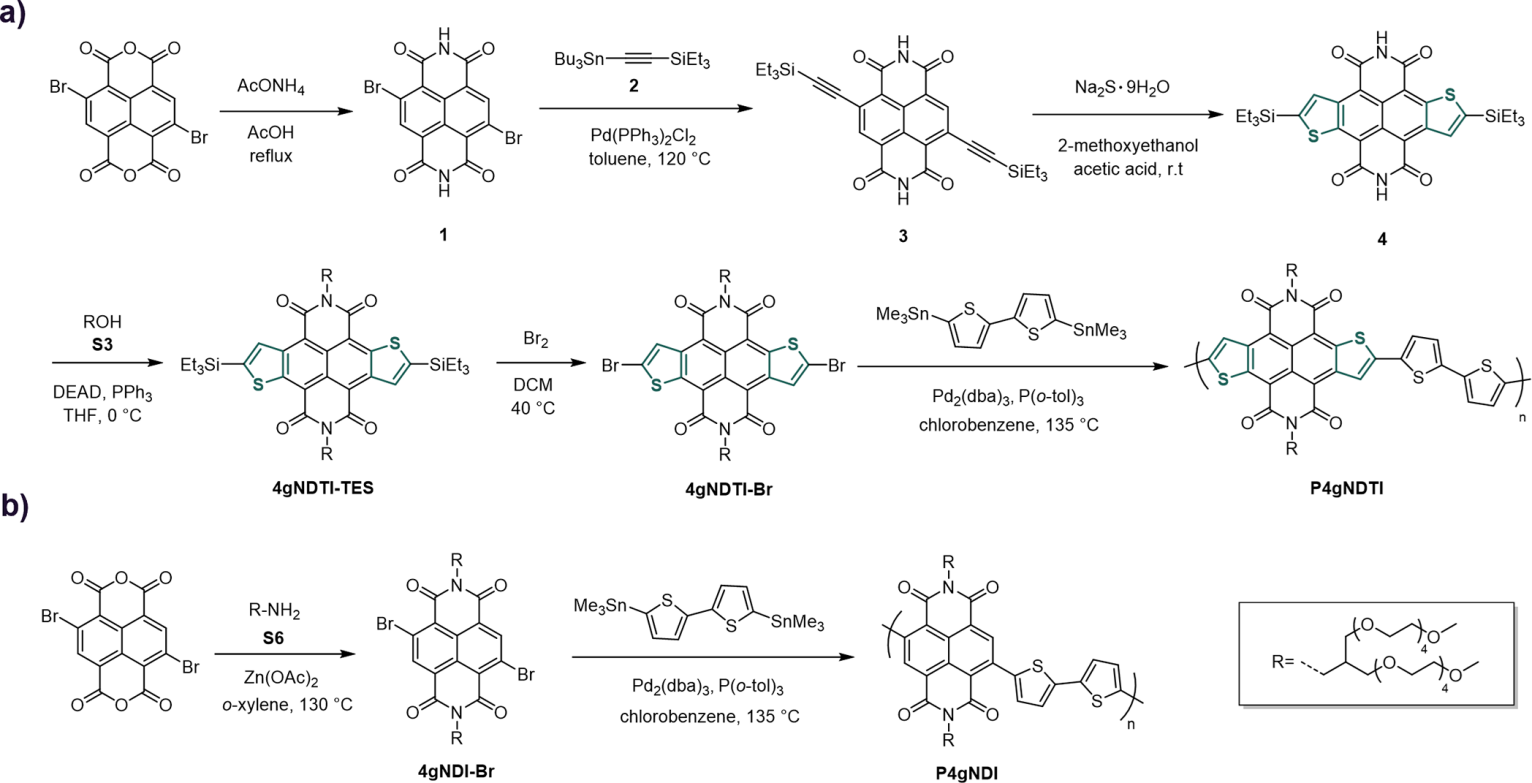
Synthesis and Chemical Structures of (a) **P4gNDTI** and (b) **P4gNDI**

The synthetic route to **P4gNDI** and **P4gNDTI** is shown in Scheme 1. The key intermediate toward **P4gNDTI** is the *N*,*N*′-unsubstituted NDTI unit **4**, which was prepared in three steps from 2,6-dibromonaphtaleneanhydride as previously reported.^38^ A Mitsunobu reaction of **4** with alcohol **S3** in the presence of diethylazodicarboxylate and triphenylphosphine was employed to introduce the branched tetraethylene glycol substituents at the imide position. Lastly, the triethylsilyl groups at the thiophene α-positions of **4gNDTI-TES** were converted to bromo groups to give the **4gNDTI-Br** monomer.

To synthetize the NDI analogue, alcohol **S3** was converted to the amine derivative using a sequence of tosylation, conversion to an azide, and Staudinger reduction, as detailed in the Supporting Information. Following a previously optimized protocol,^33^ an imidization reaction of 2,6-dibromonaphthaleneanhydride with the amine side chain **S6** and anhydrous zinc acetate as a Lewis acid catalyst afforded **4gNDI-Br** (Scheme 1b).

Having successfully synthesized the monomers, **4gNDTI-Br** and **4gNDI-Br** were subjected to Stille cross-coupling polymerizations with 5,5′-bis(trimethylstannyl)-2,2′-bithiophene to afford **P4gNDTI** and **P4gNDI**, as shown in Scheme 1. **P4gNDI** showed good solubility in acetone, ethyl acetate, tetrahydrofuran, and chloroform, while **P4gNDTI** was only soluble in chloroform at room temperature. Gel permeation chromatography was employed to estimate the molecular weights of the polymer series, using polystyrene standards and chloroform as the eluent at 40 °C, as summarized in Table 1. The number-average molecular weights (*M*_n_) and dispersities (*Đ*) were found to be 22.0 kDa (*Đ* 1.63) for **P4gNDI** and 59.4 kDa (*Đ* 4.54) for **P4gNDTI**, respectively. Although the *M*_n_ of **P4gNDTI** appears to be higher than its NDI analogue, the polymer showed a bimodal molecular weight distribution most likely due to aggregation in solution, which could have led to an overestimation of the molecular weight (Figure S1). Therefore, the *M*_n_ of the two polymers is not expected to be a large contributing factor to any differences in charge transport properties and overall device performance. It should be noted that attempts to synthetize derivatives of **P4gNDTI** with alternative side chains such as a linear heptakis(ethyelene glycol) or a shorter branched triethylene glycol chain led to the formation of highly insoluble residues or oligomeric species only. The low solubility of the NDTI-based polymers is most likely due to the increased size of the aromatic core relative to NDI, where soluble NDI polymers with either linear or shorter branched EG-based side chains have been previously synthesized in reasonably high molecular weights.^33,39^ Both **P4gNDI** and **P4gNDTI** displayed good thermal stability with 5% weight-loss temperatures of 391 and 381 °C, respectively, under nitrogen flow (Figure S2).

**Table 1:** Properties of the Polymers

polymer	*M*_n_ (kDa) [*Đ*]^a^	*E*_g_^opt^ (eV)^b^	λ_max, soln_ (nm)^c^	EA (eV)^d^	IP (eV)^e^	*d*_010_ (Å)^f^
**P4gNDI**	22.0 [1.63]	1.39	612	4.04	5.40	4.09
**P4gNDTI**	59.4 [4.54]^g^	1.30	793	4.17	5.14	3.67

a Number-average molecular weight and dispersity (GPC vs polystyrene standards in chloroform at 40 °C).

b Optical band gap estimated from thin film absorption onset.

c Solution absorption spectra (chloroform).

d Cyclic voltammetry of polymer thin films on ITO-coated glass substrates in acetonitrile with 0.1 M tetrabutylammonium hexafluorophosphate as the supporting electrolyte (Figure S7).

e Measured by photoelectron spectroscopy in air.

f Out-of-plane π-stack scattering peak *d*-spacing determined by GIWAXS.

g Bimodal distribution observed.

To investigate the backbone conformation of the polymers, DFT calculations were carried out for methyl-functionalized tetramers with an optimally tuned ωB97XD functional and 6-31G* basis set (geometry optimizations), as well as a B3LYP-D3 functional and 6-31G* basis set (torsional energy profiles). As illustrated in Figure S3, the **P4gNDI** backbone shows a significant twist and dihedral angles of 49–59° between the NDI and bithiophene moieties, whereas the **P4gNDTI** backbone adopts a highly coplanar geometry with small dihedral angles of 3–5° for the NDTI-T2 linkage. Further insight into the torsional disorder of the polymer backbone was provided by calculating potential energy surfaces along the dihedral coordinate connecting a thiophene ring with either NDI or NDTI units (Figure S4). In agreement with previous reports,^40^ the NDI-T unit shows a double well potential with stable conformations at 50° and 130°. In sharp contrast, the NDTI-T unit shows an energetic minimum at 180° and a high barrier for rotation of 5.3 kcal/mol, corroborating the enhanced backbone coplanarity upon extension of the NDI core size to a NDTI unit.

The UV–Vis absorption spectra of the polymers as a thin film and in solution are shown in the Supporting Information (Figure S5), and the corresponding optical parameters are summarized in Table 1. As expected,^41^**P4gNDI** shows two absorption features, with a high energy band, ascribed to the π–π* transition (λ_max_ ≈ 400 nm) and a lower energy band, due to intramolecular charge transfer (ICT, *λ*_max_ ≈ 715 nm). **P4gNDTI** shows an additional weak band between 500 and 590 nm and markedly red-shifted absorption features compared to their NDI analogues, extending into the near-IR region. Replacing the NDI acceptors with NDTI units led to a decrease in the optical band gap from 1.39 to 1.30 eV, consistent with extended π-conjugation for the more coplanar **P4gNDTI** backbone. Photoelectron spectroscopy in air measurements were employed to determine the ionization potentials (IP) of the polymers, while the electron affinities (EA) were determined using cyclic voltammetry (CV) of the polymer thin films. **P4gNDTI** shows a simultaneous decrease in the IP and increase in the EA relative to its NDI analogue. In line with previous reports,^37,42^ introduction of NDTI units led to a stabilization of the LUMO levels, with EA of 4.17 and 4.04 eV for **P4gNDTI** and **P4gNDI**, respectively (Figure S6).

**Figure 1:**
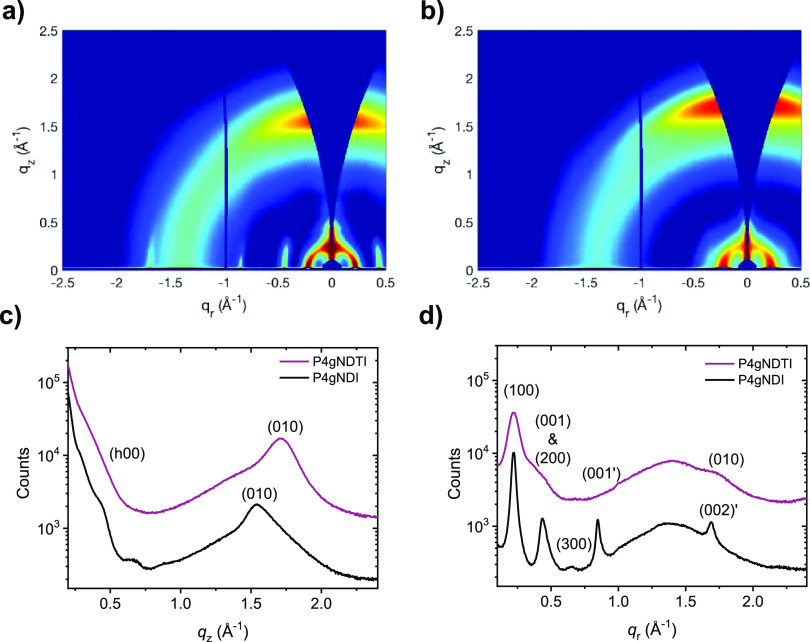
GIWAXS of as-cast films: *q*_r_–*q*_z_ maps of scattered X-ray intensity from thin films of (a) **P4gNDI**, (b) **P4gNDTI**, and (c) out-of-plane (*q*_z_) and (d) in-plane (*q*_r_) line cuts of each polymer.

The effect of backbone coplanarity on the structural organization of the polymer thin films was investigated by grazing incidence wide-angle X-ray scattering (GIWAXS) measurements (Figure 1 and Table S1). All polymers adopt a predominantly face-on orientation, with enhanced out-of-plane π-stack scattering and in-plane lamellar scattering. The nature of the polymer backbone does not appear to affect side chain ordering, with both **P4gNDI** and **P4gNDTI** displaying a lamellar *d*-spacing of 28.4 Å. As previously demonstrated for NDI-T2 polymers,^43^**P4gNDI** display in-plane backbone scattering peaks associated with two distinct polymorphs of cofacial alignment in the backbone direction of adjacent π-stacked chains: Form I [noted as (001)] and Form II [noted as (001)′ and (002)′].^43^ These two forms, I and II, represent the cofacially aligned π–π stacking of NDI on NDI (and T2 on T2) and NDI on T2 (and T2 on NDI), respectively. In contrast, **P4gNDTI** displayed no clear in-plane (001) peak, and (001)′ and (002)′ peaks were absent, indicating no similar preferred cofacial alignment. Notably, introduction of NDTI units leads to tighter π–π stacking of the polymer chains with a significant contraction of the out-of-plane π-stack *d*-spacing (∼0.40 Å) of **P4gNDTI** relative to its NDI analogue. This seems to indicate a trade-off between cofacial alignment in the backbone direction between π-stacked chains and the tightness of π-stacking spacing. In light of the computational assessment of planarity, it is rationalized that the large dihedral angles along the backbone of the **P4gNDI** drive discrete cofacial alignment in the solid state and frustrates close π–π stacking, whereas the highly planar **P4gNDTI** shows no preference in cofacial alignment, allowing for stronger intermolecular π–π interactions (tighter *d*-spacing), consistent with the observed optoelectronic properties (Table 1).

**Figure 2:**
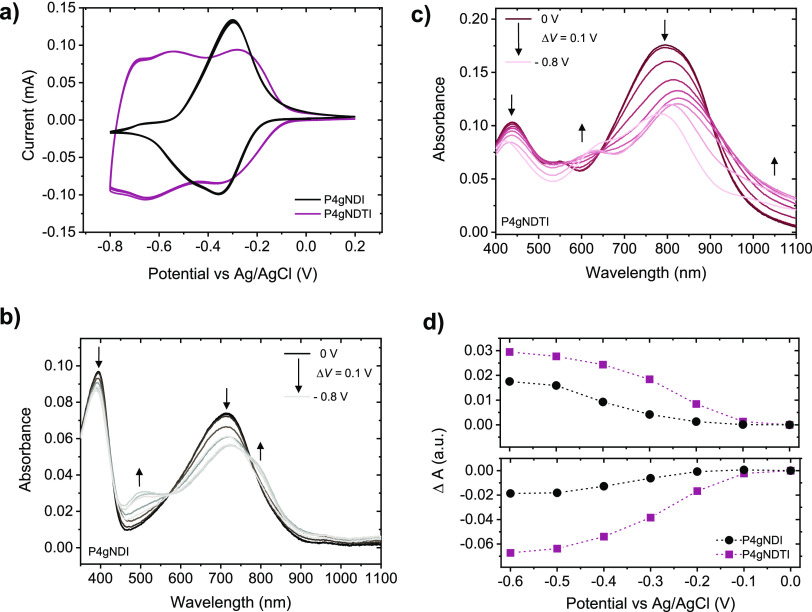
(a) Three-electrode cyclic voltammetry measurement of **P4gNDI** and **P4gNDTI** on glassy carbon electrodes in N_2_-saturated 0.1 M NaCl aqueous solution with a scan rate of 100 mV s^–1^, applying potentials vs a Ag/AgCl reference electrode (scans 2–4). Evolution of the UV–Vis absorption spectrum during charging between 0 and −0.8 V vs Ag/AgCl for (b) **P4gNDI** and (c) **P4gNDTI** in a N_2_-saturated 0.1 M NaCl aqueous solution in steps of 100 mV. (d) Relative difference between the absorbance recorded with bias (0 to −0.6 V versus Ag/AgCl) and the initial spectrum without bias of the polaron peak (502 nm for **P4gNDI** and 1048 nm for **P4gNDTI**) and neutral polymer ICT peak (696 nm for **P4gNDI** and 760 nm for **P4gNDTI**).

The electrochemical doping processes in aqueous electrolytes were investigated by carrying out CV and spectroelectrochemical measurements of the polymer films when immersed in 0.1 M NaCl aqueous solutions. Figure 2 shows the electrochemical reduction of the polymer series for three consecutive doping cycles. The high electron affinity and ease of electrochemical reduction in aqueous media of **P4gNDTI** were corroborated by its low reduction onset of −0.08 vs Ag/AgCl in a 0.1 M NaCl aqueous solution, considerably shifted to more positive values when compared with **P4gNDI** (−0.16 V vs Ag/AgCl). While **P4gNDI** shows a well-resolved reduction peak characteristic for NDI-T2 polymers with hydrophilic side chains,^32^**P4gNDTI** displays broader reduction waves, where two peaks can be observed. We assign the peaks to formation of the electron polaron and electron bipolaron of NDTI, where new absorption features are observed for the NDTI polymer when the bipolaron is formed, in agreement with previous findings for NDI-based polymers.^32^ Notably, **P4gNDTI** also demonstrates improved stability during continuous cycling, showing stable current for the first reduction peak and retention of 94% of the initial current of the second peak, relative to 74% retention for **P4gNDI** after 50 charging and discharging cycles (Figure S7).

The optical changes of the polymer thin films upon electrochemical reduction are shown in Figure 2. At potentials between 0 and −0.6 V vs Ag/AgCl, **P4gNDI** shows a gradual decrease in the absorption of the π–π* and ICT bands with concomitant formation of a new feature at 500 nm and a shoulder at 791 nm, which is consistent with formation of a radical anion (polaron) species (Figure 2).^44^ In the case of **P4gNDTI**, the absorption of the bands at 438, 552, and 794 nm gradually decreases, while a new feature emerges at 609 nm and the absorption in the sub-band gap region increases (Figure 2). When monitoring the relative changes in the intensity of the ICT band as a function of applied bias, the spectral changes start occurring at a lower potential for **P4gNDTI** compared to **P4gNDI**, indicating an earlier doping onset. Upon applying potentials more negative than −0.8 V vs Ag/AgCl, **P4gNDTI** shows evidence of transformation into a distinct species with new absorption features at 422, 630, and 783 nm, most likely due to formation of the bipolaron species. When the potential is reversed to 0 V, the spectrum of the neutral polymer is restored in shape and intensity, demonstrating electrochemical reversibility of the observed charging processes (Figure S8). On the other hand, the characteristic spectroscopic features of the bipolaron could not be identified for **P4gNDI**, in agreement with previous studies of NDI-T2 polymers functionalized with EG side chains.^8,34^

Several studies have demonstrated a strong correlation between the polymer microstructure, and its swelling ability in aqueous environments.^17,31,45^ To study the effect of backbone coplanarity on the uptake of water and ions during electrochemical doping, electrochemical quartz crystal microbalance with dissipation monitoring (eQCM-D) measurements were performed for **P4gNDI** and **P4gNDTI**. The QCM-D sensors were first analyzed prior to biasing in order to determine the extent of passive swelling in contact with a 0.1 M NaCl aqueous solution (Figure S9). Upon electrolyte exposure, **P4gNDI** showed a surprisingly large mass uptake, with a passive swelling that could not be quantified within the measurement window of the instrument. This transition into a hydrogel-like state has been shown for other NDI-based polymers,^32^ although the transition typically occurs at high doping potentials, whereas **P4gNDI** appeared to transition spontaneously without any applied bias. In contrast, a **P4gNDTI** thin film with a comparable thickness showed a passive swelling of 57% of its initial mass, indicating lower extent of swelling upon substituting the NDI with NDTI units. During electrochemical reduction in aqueous electrolytes, **P4gNDTI** shows further mass uptake with an additional 24% at −0.4 V and 218% at −0.7 V versus Ag/AgCl (Figure S10), as more hydrated ions are injected into the film to charge compensate the electronic charge carrier on the reduced polymer backbone.

**Figure 3:**
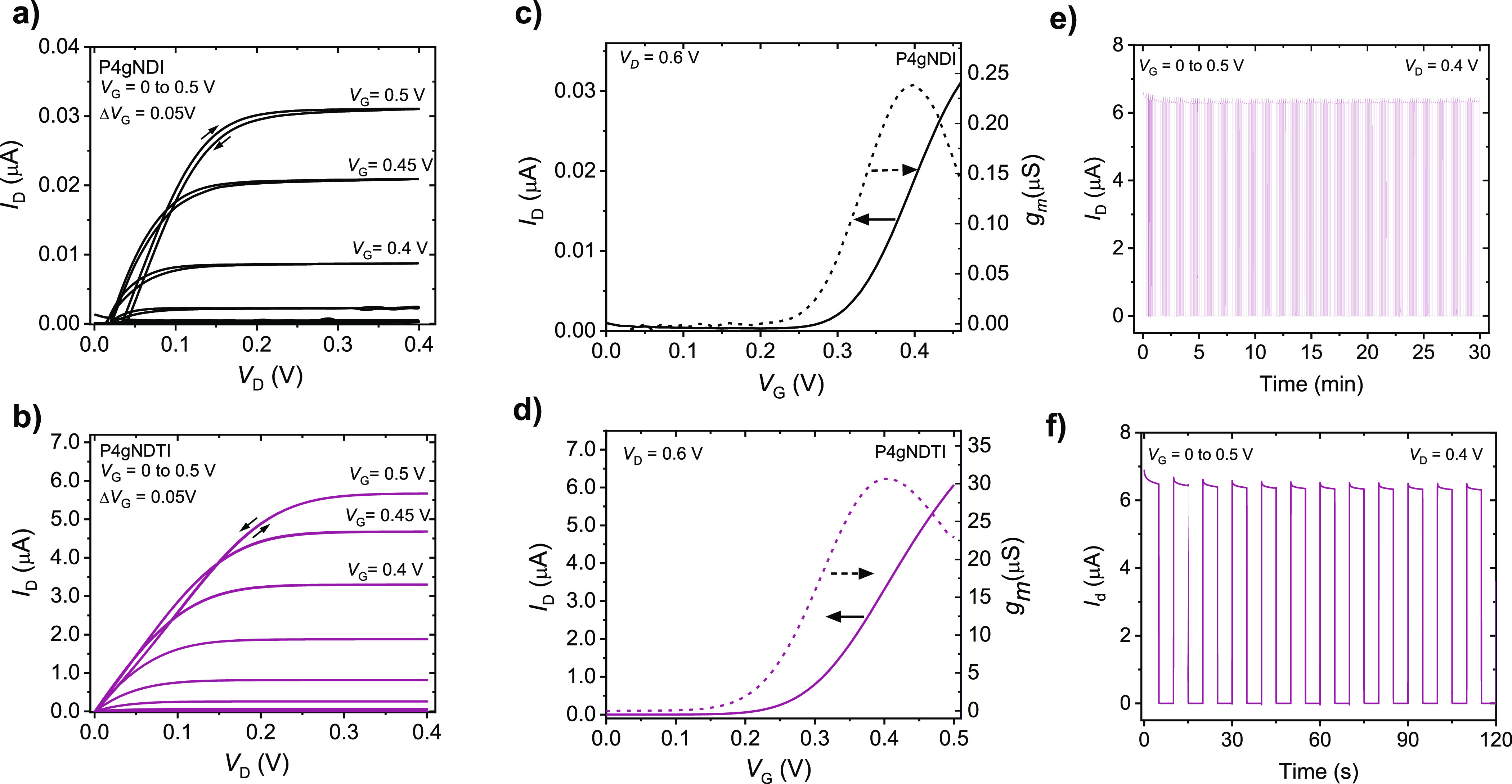
Performance of **P4gNDI** (top, *d* = 1.26 ± 0.10 μm) and **P4gNDTI** (bottom, *d* = 0.59 ± 0.12 μm) OECT devices (*W* = 100 μm, *L* = 10 μm, 200 mV/s) showing (a,b) output curves for 0 < *V*_G_ < 0.5 V with Δ*V*_G_ = 0.05 V (c,d) transfer curves at *V*_D_ = 0.6 V and corresponding transconductance. Stability pulsing measurements with alternating gate potentials between *V*_G_ = 0 V and *V_G_* = 0.5 V for **P4gNDTI** with a pulse duration of 5 s for OECT channels biased at *V*_D_ = 0.4 V for (e) 30 min and (f) first few switching cycles. All measurements were performed in ambient conditions in 0.1 M NaCl with a Ag/AgCl pellet gate electrode.

To gain insight into the mixed ionic-electronic conduction properties, OECT devices comprising the two polymers as the channel material were investigated in aqueous electrolytes. The output and transfer characteristics (Figures 3 and S11) demonstrate enhancement-mode n-type operation for both polymers. Consistent with previous reports of NDI-T2 copolymers with high densities of polar side chains,^33,34^**P4gNDI** shows non-ideal OECT characteristics, with forward–reverse hysteresis and an irreversible degradation of the drain current at gate voltages exceeding 0.45 V (Figures 3a and S11a). In contrast, **P4gNDTI** devices show minimal hysteresis for the forward and reverse voltage scans, indicating more reversible electrochemical charging/discharging processes. In line with its higher electron affinity and lower reduction onsets, the **P4gNDTI**-based devices are characterized by lower threshold voltages of 0.20 ± 0.003 V than **P4gNDI** (0.27 ± 0.05 V). Interestingly, upon monitoring the evolution the gate current (*I*_G_) during operation of the OECTs in ambient conditions, it was found that the onset for increased *I*_G_ is shifted to higher potential values when going from **Pg4NDI** to **P4gNDTI**. This suggests that by increasing the backbone coplanarity and electron affinity of the n-type polymer, the OECT operation is shifted into a potential range where fewer parasitic side reactions of the reduced polymer with molecular oxygen (i.e., ORR) are suppressed, as evidenced by a lower gate current when operating the OECT in ambient conditions (Figure S12).

Based on the OECT transfer characteristics (Figure 3), the device performance was found to increase by more than two orders of magnitude upon enhancing the coplanarity of the backbone, reaching a thickness-normalized *g*_m_ of 0.47 ± 0.06 S cm^–1^ for **P4gNDTI** (Figure 3, Table 2). To allow an evaluation of the material-dependent properties, the mobility capacitance (μC*) product corresponding to the peak *g*_m_ was extracted and found to increase similarly from **P4gNDI** [(1.61 ± 0.46) × 10^–3^ F V^–1^ cm^–1^ s^–1^] to **P4gNDTI** (0.27 ± 0.04 F V^–1^ cm^–1^ s^–1^). To understand the origin of the improved μC* of **P4gNDTI**, electrochemical impedance spectroscopy (EIS) measurements were performed under a range of offset potentials to extract the capacitance (C*) of the polymers (Figure S13). **P4gNDI** exhibits a C* of 219 F cm^–3^, which decreases slightly upon backbone modification to 167 ± 11.4 F cm^–3^ for **P4gNDTI**. The slight decrease in C* implies that the improved overall device performance and μC* of **P4gNDTI** is likely due to enhanced charge transport properties and a superior electron mobility (μ) in OECTs. The electron mobility was extracted from the OECT characteristics in the saturation regime and found to increase by more than two orders of magnitude from **P4gNDI** [(7.34 ± 2.11) × 10^–6^ cm V^–1^ s^–1^] to **P4gNDTI** [(1.42 ± 0.22) × 10^–3^ cm V^–1^ s ^–1^]. The superior electronic charge transport properties of **P4gNDTI** are attributed to improved planarity and tighter π–π stacking in solid state compared to **P4gNDI**. Given the distinct swelling behavior observed by eQCM-D, **P4gNDTI** is more likely to preserve the integrity of its film microstructure and crystallite interconnectivity during operation in aqueous electrolyte due to a more controlled swelling and lower uptake of hydrated species.^12,34^ The electron mobility of **P4gNDTI** is on the same order of magnitude as the highest reported values for previous n-type OECT materials,^28^ comparable to BBL^13^ and p(C6-T2),^35^ with only few values superseding this, such as p(g_7_NC_10_N), with a reported mobility of 1.20 × 10^–2^ cm^2^ V^–1^ s^–1^.^46^ This demonstrates that enhancing the backbone coplanarity is an effective strategy to improve the charge transport properties of n-type polymers.

**Table 2:** OECT Parameters of the Polymers

polymer	*g*_m_ (S cm^–1^)^a^	*V*_T_ (V)^b^	[μ_el_C*] (F cm^–1^ V^–1^ s^–1^)^c^	C* (F cm^–3^)^d^	*μ*_el_ OECT (cm^2^ V^–1^ s^–1^)^e^	*I*_ON_/*I*_OFF_^f^
**P4gNDI**	(1.87 ± 0.25) × 10^–3^	0.27 ± 0.05	(1.61 ± 0.46) × 10^–3^	219	(7.34 ± 2.11) × 10^–6^	20.2 ± 0.15
**P4gNDTI**	0.47 ± 0.06	0.20 ± 0.003	0.27 ± 0.04	167 ± 11.4	(1.42 ± 0.22) × 10^–3^	(4.18 ± 1.1) × 10^3^

a OECT peak transconductance measured at *V*_G_ = 0.4 V normalized by thickness.

b Threshold voltage extracted from the √*I*_D_ versus *V*_G_ plot.

c [μC*] estimated from the transfer characteristics of the OECT with known channel dimensions and biasing conditions.

d Determined from the electrochemical impedance spectra of the polymers coated on Au electrodes (600 × 600 μm) in a 0.1 M NaCl aqueous solution (*V* = −0.4 V versus Ag/AgCl).

e Electron mobility extracted from the OECT transfer characteristics in the saturation regime.

f ON/OFF current ratio with *I*_ON_ measured at *V*_G_ = 0.4 V.

The shelf-life stability of **P4gNDTI** and **P4gNDI** in OECTs was investigated using aging studies of the devices over a period of 4 weeks, while they were stored in ambient conditions and without continual exposure to electrolyte. We found that **4gNDTI**-based devices had a marked improvement in stability, when directly compared with the **P4gNDI-**based devices. For **4gNDTI**, we observed an improvement in the transconductance by ≈11% between Week 0 and Week 1, from 2.37 μS to 2.65 μS at *V*_G_ = 0.45 V, following a decrease in the off-current (*I*_OFF_), from 2.31 × 10^–3^ μA to 8.49 × 10^–4^ μA at *V*_G_ = 0.1 V. From Week 2 onward, the maximum on-current (*I*_ON_) (Figure S14a) for the **P4gNDTI** OECTs is found to be stable throughout the duration of the 4-week aging test, corresponding to a negligible decrease in transconductance (Figure S14b). A similar behavior is observed with the **P4gNDI** devices in terms of a decrease in *I*_OFF_ between Week 0 and Week 1. However, in contrast to the **P4gNDTI** OECTs, the **P4gNDI** device performance is not stable for the duration of the month-long test. Specifically, both the *I*_ON_ and transconductance show marked decreases, where *I*_ON_ decreases by ≈61%, from 0.114 μA to 0.070 μA between Week 2 and Week 3, with a subsequent, clear decrease from 0.070 μA to 0.056 μA between Week 3 and Week 4. Overall, the **P4gNDTI** polymer not only shows significantly improved transistor characteristics, but also demonstrates superior shelf-life stability.

Next, the stability during OECT operation of the polymers was investigated by monitoring the drain current upon applying successive gate voltage steps of Δ*V*_G_ = 0.5 V (Figure 3e). While **P4gNDI** proved impractical for long-term operation due to its low ON current, **P4gNDTI** shows a remarkably stable current response. After a drop by ≈6% after the first switching cycle, **P4gNDTI** shows no change in the drain current after 30 min of ON–OFF switching in aqueous media. The stable current response of **P4gNDTI** is comparable to the side-chain free rigid polymer BBL^13^ and improved compared with previously reported n-type polymers based on fused lactam units,^36,47^ which, although displaying higher μC*, show a degradation of the ON current within minutes of OECT operation in aqueous electrolytes. Therefore, this study demonstrates that the NDTI unit is a promising acceptor block for achieving a high operational stability, a key metric when benchmarking materials and a requirement for their successful integration in OECT-based technologies operating in aqueous electrolytes. We envisage that combining this approach to promote backbone coplanarity with recently reported side chain engineering strategies^22,34,35^ offers great potential for further improving the stability and performance of n-type OECT materials.

## Conclusions

In this work, we investigate the effect of enhanced backbone coplanarity on the electrochemical activity and OECT characteristics of n-type polymers by introducing NDTI units within the polymer backbone, which promote a more coplanar geometry with lower torsional disorder relative to the previously employed NDI units. Our findings show that incorporation of NDTI units leads to a higher electron affinity and reversible electrochemical reduction at a more positive potential, coupled with a lower swelling ability in aqueous media. We demonstrate that enhancing backbone coplanarity leads to an increase of more than two orders of magnitude in the thickness-normalized OECT transconductance, with an electron mobility of (1.42 ± 0.22) × 10^–3^ cm^2^ V^–1^ s^–1^ and a μC* of 0.27 ± 0.04 F cm^–1^ V^–1^ s^–1^ for the NDTI-based copolymer. The NDTI-based polymer also demonstrates a negligible decrease in both the maximum on-current and transconductance in a 4-week aging test. Furthermore, the NDTI-based polymer is able to preserve its superior mixed ionic-electronic conduction properties during extended OECT operation and shows a stable current response after 30 min of ON–OFF switching in aqueous electrolytes. Our results reveal a strong correlation between the backbone coplanarity of n-type polymers and their performance in OECTs, expanding the synthetic toolbox available for developing next-generation OECT materials.

## Experimental Section

### Synthesis and Characterization of the Polymers

The procedures for the synthesis and characterization of the polymers are described in the Supporting Information.

### Grazing Incidence Wide Angle X-Ray Scattering

GIWAXS measurements were carried out at the Advanced Photon Source at Argonne National Laboratory on beam line 8-ID-E at room temperature under vacuum with 10.92 keV (λ = 1.135 Å) synchrotron radiation with a 0.14° incident angle and measured with a Pilatus 1 M hybrid pixel array detector during 10–20 s exposures. The 2D GIWXAXS patterns were collected from films spin-coated (600 rpm for 60 s) from chloroform (5 mg mL^–1^) on Si wafer substrates (University Wafer). Multiple exposures were averaged to create 2D images. Averaged images with different detector z-positions were stitched together to fill the vertical gaps between detector chips. Data analysis was carried out with a GIXSGUI Matlab toolbox^48^ and custom curve fitting code.

### Electrochemical Analysis

Cyclic voltammograms were recorded using a standard three-electrode setup containing a polymer-coated glassy carbon electrode, a platinum mesh counter electrode (active area 25 × 35 mm), and a Ag/AgCl reference electrode (3 M NaCl/H_2_O), connected to a Metrohm Autolab PGSTAT101 potentiostat. The polymers were deposited on the glassy carbon electrode by drop-casting from chloroform solutions (5 mg mL^–1^). The measurements were carried out in either an anhydrous 0.1 M acetonitrile solution of tetrabutylammonium hexafluorophosphate (TBAPF_6_) or a 0.1 M NaCl aqueous solution as the supporting electrolyte at a scan rate of 100 mV s^–1^ under a nitrogen blanket. The electrolyte solutions were degassed with nitrogen for 15 min prior to the measurements.

The spectroelectrochemical measurements were performed in a 0.1 M NaCl aqueous solution using a similar three-electrode setup, with ITO substrates as the working electrode, a platinum mesh counter electrode (active area 25 × 35 mm), and a Ag/AgCl reference electrode. The polymers were deposited by spin-coating chloroform solutions (5 mg/mL) at 1500 rpm on ITO-coated glass slides. The substrates were immersed in a custom-made cell containing the electrolyte solution, which was positioned in the beam path of a UV-1601 Shimadzu UV–Vis spectrometer. Each potential was applied for 5 s before recording the optical spectra to allow the films to equilibrate.

EIS was performed using polymer films cast on gold substrates (600 × 600 μm) as the working electrode, a platinum mesh as the counter, and an Ag/AgCl reference electrode coupled to a potentiostat (Metrohm Autolab). The measurements were carried out in a 0.1 M NaCl aqueous solution in ambient conditions. The measurements were carried out with a 10 mV sine wave and frequencies from 0.1 Hz to 10 kHz. Analysis was performed with Metrohm NOVA software and custom MATLAB tools.

### Electrochemical Quartz Crystal Microbalance with Dissipation Monitoring

eQCM-D measurements were performed using a Q-sense analyzer (QE401, Biolin Scientific). Passive swelling measurements of the polymer films in a 0.1 M NaCl aqueous solution (Figure S9) were performed as previously described.^19,49^ The QCM-D response of the bare Au sensors was recorded first in air, followed by the injection of the 0.1 M NaCl aqueous solutions into the chamber. This resulted in large shifts in frequency (*f*) and dissipation of energy (*D*), due to the density differences between the two media. The measurements were then stopped, the sensors were removed, and polymer films were spun cast directly on the same sensor from a 5 mg/mL chloroform solution at 1500 rpm. The absolute *f* value for each polymer-coated sensor was obtained both in air and in a 0.1 M NaCl aqueous solution after the *f* signal was perfectly flat (i.e., *f* < 0.5 Hz) to ensure that the system is in equilibrium. The absolute difference in *f* for multiple overtones between the bare sensor and the polymer-coated sensors, both in air and in the 0.1 M NaCl aqueous solution, was compared using the function “stitched data” of Q-soft software. This function compared the selected data sets based on the raw frequencies measured and excluded the effect of the different densities between the two mediums. Thus, the difference of the *f* values of the stitched data is directly analogous to the thickness of the polymer in both media, which is calculated by using the Sauerbrey equation:
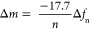
1

eQCM-D measurements were performed using an Autolab PGstat128N potentiostat coupled with the Q sense electrochemistry module comprising the three-electrode setup. The three-electrode setup consisted of a Ag/AgCl reference, Pt counter, and Au/polymer eQCM-D sensor as the working electrode with an electrochemical area of 0.7854 cm^2^. Since the films become soft and uptake a significant amount of water under doping potentials, the Kelvin–Voigt viscoelastic model was used to fit the data. A Kelvin–Voigt element has a complex shear modulus as described below:
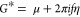
2where G* is the complex shear modulus, μ is elasticity (kgm^–1^ s^–2^), η is viscosity (kg m^–1^ s^–1^), and *f* is the frequency.

To quantify the mass correctly, the *f* and *D* data of three different overtones (3rd, 5th, and 7th) were used. The good quality of the fits guaranteed the accurate mass calculation accumulated within the films upon applied potentials. The modeling and data analysis was carried out using Q-Tools and D-find software.

### OECT Fabrication and Characterization

The OECTs were fabricated using previously reported photolithographic procedures, with gold contacts and a Parylene C insulating layer.^18,50^ The channels used in this work had a width of 100 μm and length of 10 μm, with the exception of the devices used for aging measurements (Figure S14), which had a channel length of 30 μm and a width of 600 μm. The polymer solutions (5 mg mL^–1^) were drop-cast before sacrificial peel off of Parylene C. All measurements were performed using an external Ag/AgCl pellet electrode as the gate in a 0.1 M NaCl aqueous solution in ambient conditions. The *I*–*V* characteristics were recorded using a dual-channel source-meter unit (NI-PXI) with a custom-written control code in LabVIEW.

### OECT Aging Measurements

Each of the NDI and NDTI polymer solutions was prepared at 5 mg mL^–1^ in chloroform with 0.1 vol % methanol and spin-coated onto the OECT substrates at 700 rpm for 40 s. Shelf-life stability for the OECTs was measured by observing the *I*–*V* characteristics in the same manner at regular 1-week intervals, over the course of 4 weeks, where the electrical properties of the OECTs were characterized using a Keysight B2912B Source Measurement Unit, while exposing to 0.1 M NaCl electrolyte solution and using Ag/AgCl as the gate electrode. A Dektak Profilometer was used to measure film thickness in the channel of the devices.

## Supporting Information Available

Monomer and polymer synthesis details, GPC measurements, thermogravimetric analysis, DFT calculations, UV–Vis measurements, GIWAXS analysis, cyclic voltammetry measurements, spectroelectrochemistry measurements, QCM-D and eQCM-D measurements, OECT measurements, EIS measurements, and device stability measurements (PDF)
