# Supplementary material for: Enhancing the Backbone Coplanarity of n-Type Copolymers for Higher Electron Mobility and Stability in Organic Electrochemical Transistors
Source: Chem Mater. 2022 Sep 27;34(19):8593–602. doi: 10.1021/acs.chemmater.2c01552 (PMC9558307; doi:10.1021/acs.chemmater.2c01552)
Supplement: Supplementary file 1 — cm2c01552_si_001.pdf [file cm2c01552_si_001.pdf]

Supporting Information

Enhancing the backbone coplanarity of n-type copolymers for higher electron mobility and stability in organic electrochemical transistors

Iuliana P. Maria*,[1,2] Sophie Griggs,[2] Reem B. Rashid,[3] Bryan D. Paulsen,[3] Jokubas Surgailis,[4] Karl Thorley,[5] Vianna N. Le[6], George T. Harrison,[7] Craig Combe,[7] Rawad Hallani,[7] Alexander Giovannitti,[8] Alexandra F. Paterson,[6] Sahika Inal,[4] Jonathan Rivnay,[3,9] Iain McCulloch* [2,7]

[1] Department of Chemistry and Centre for Plastic Electronics, Imperial College London, London SW7 2AZ, United Kingdom.

[2] Department of Chemistry, Chemistry Research Laboratory, University of Oxford, Oxford, OX1 3TA, United Kingdom.

[3] Department of Biomedical Engineering, Northwestern University, IL 60208, USA.

[4] Biological and Environmental Science and Engineering Division, King Abdullah University of Science and Technology, Thuwal 23955-6900, Saudi Arabia.

[5] Department of Chemistry, University of Kentucky, Lexington, Kentucky 40506-0055, USA.

[6] Department of Chemical and Materials Engineering, University of Kentucky, Lexington, Kentucky, 40506-0055, USA.

[7] King Abdullah University of Science and Technology, KAUST Solar Center, Thuwal 23955-6900, Saudi Arabia.

[8] Department of Materials Science and Engineering, Stanford University, Stanford, CA 94305, USA.

[9] Simpson Querrey Institute, Northwestern University, IL 60611, USA.

Contents

Methods **3**

Synthetic procedures **4**

Synthesis of the branched side chains 4

Synthesis of 4gNDTI-Br 8

Synthesis of 4gNDI-Br 12

Synthesis of the polymers 13

GPC measurements **15**

Thermogravimetric analysis **16**

DFT calculations **17**

UV-Vis measurements **18**

GIWAXS analysis **18**

Cyclic voltammetry in organic electrolytes **19**

Spectroelectrochemistry measurements **20**

QCM-D measurements **21**

EQCM-D measurements **22**

OECT measurements **23**

EIS measurements **25**

Stability of the OECT devices **26**

NMR Spectra **27**

References **37**

Methods

Materials characterization. ^{1}H NMR and ^{13}C NMR spectra were recorded on 400 MHz and 100 MHz Bruker spectrometers, respectively, at ambient temperature unless otherwise stated. Chemical shifts are reported in units of parts per million (ppm, δ) for solutions in chloroform-*d* and coupling constants (J) are given in Hz. The chemical shifts were referenced using the residual chloroform (^{1}H NMR: 7.26 ppm, ^{13}C NMR: 77.36 ppm) or DMSO (^{1}H NMR: 2.54 ppm, ^{13}C NMR: 40.45 ppm) peaks as internal standards. Electrospray (ES-ToF) mass spectrometry was performed using a Micromass LCT Premier mass spectrometer. Number-average (M_n) and weight-average (M_w) molecular weights were determined using an Agilent Technologies 1260 infinity GPC at 40 °C in chloroform, using two PLgel 10 micrometer Mixed-B columns in series (300 × 7.5 mm), and calibrated against narrow dispersity ($Đ$ < 1.10) polystyrene standards. Thermogravimetric analysis (TGA) analysis was carried out on a Mettler Toledo instrument, heating from 30.0 °C to 105.0 °C (34.99 K min^{-1}), keeping the temperature at 105.0 °C for 10.0 min and heating from 105.0 to 800.0 °C (10.0 K min^{-1}) under nitrogen flow (50 mL min^{-1}).

Optical characterization. UV-Vis absorption spectra were recorded on UV-1601 Shimadzu UV-Vis spectrometer for both chloroform solutions and thin films at ambient conditions. The samples for solid state measurements were prepared by spin coating polymer solutions in chloroform (5 mg mL^{-1}) on glass substrates at 1000 rpm for 1 minute. Photoelectron spectroscopy in air (PESA) was carried out on a Riken-Keiki AC-2 PESA Spectrometer.

Synthetic procedures

General experimental. All reactions were performed in oven-dried glassware under a nitrogen atmosphere using standard Schlenk techniques, unless otherwise stated. Column chromatography was performed on Geduran® silica gel, particle size 40–63 µm or with Biotage ZIP columns, using the indicated solvents. Preparative gel permeation chromatography was performed using a LaboACE LC-5060 instrument with a separation line containing 2HR and 2.5HR columns. All commercially available materials were used as received.

Synthesis of the branched side chains

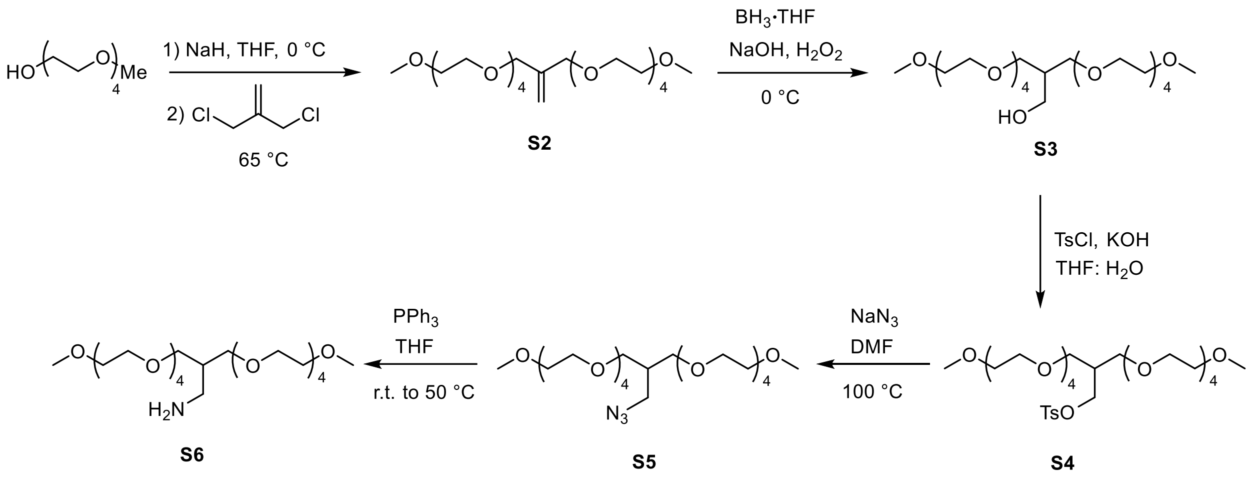

Scheme S1. Synthesis of the branched ethylene glycol-based side chains.

16-Methylene-2,5,8,11,14,18,21,24,27,30-decaoxahentriacontane (S2)

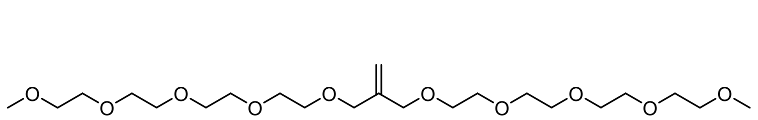

Tetraethylene glycol monomethyl ether (20.9 g, 100.4 mmol) was added dropwise to a suspension of sodium hydride (60% dispersion in mineral oil, 4.56 g, 113.9 mmol) in anhydrous THF (200 mL) at 0 °C. When gas evolution ceased, methallyl dichloride (4.18 g,

33.5 mmol, 1 equiv) was added dropwise and the resulting mixture was stirred for 15 min at 0 °C. The mixture was allowed to reach room temperature and subsequently heated to 65 °C overnight. After cooling to 0 °C, the reaction mixture was quenched with distilled water (150 mL) to quench excess sodium hydride and extracted with dichloromethane. The organic fractions were washed with distilled water, dried over anhydrous $MgSO_4$ and the solvent was removed *in vacuo*. Column chromatography (ethyl acetate: hexane, 3:2) afforded the title product (14.32 g, 91%) as a pale yellow oil. ^{1}H NMR (400 MHz, chloroform-*d*) δ 5.17 (br s, 2H), 4.02 (br s, 4H), 3.68 – 3.51 (m, 32H), 3.37 (s, 6H). ^{13}C NMR (100 MHz, chloroform-*d*) δ 142.8, 114.4, 72.2, 72.1, 71.0, 70.9 – 70.8 (multiple peaks), 69.8, 59.4. HRMS (ES-ToF): 491.2817 [M+Na$^+$] (calc. 491.2827).

13-(2,5,8,11-Tetraoxadodec-1-yl)-2,5,8,11-tetraoxatetradecan-14-ol (S3)

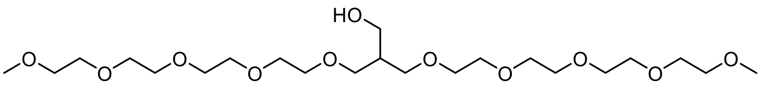

Borane tetrahydrofuran complex solution (1 M, 11.0 mL, 11.0 mmol) was added dropwise to a solution of **S2** (4.90 g, 10.5 mmol) in anhydrous THF (40 mL) at 0 °C. After stirring for 2 h at 0 °C, an aqueous solution of sodium hydroxide (2 M, 20 mL) was added dropwise and the mixture was stirred for a further 15 min at 0 °C. The mixture was slowly treated with H_2O_2 (30%, 10 mL) at 0 °C and allowed to reach room temperature and stirred for 30 min. The reaction mixture was saturated with potassium carbonate and extracted with DCM. The organic fractions were washed with distilled water and dried over anhydrous $MgSO_4$. The solvent was removed *in vacuo* and the crude product was purified using column chromatography (ethyl acetate, then ethyl acetate: methanol, 90:10) to give the title product (3.81 g, 68%) as a colourless oil. ^{1}H NMR (400 MHz, chloroform-*d*) δ 3.71 (d, *J* = 5.6 Hz, 2H), 3.66 – 3.50 (m, 36H), 3.35 (s, 6H), 2.10 (hept, *J* = 5.6 Hz, 1H). ^{13}C NMR (100 MHz, chloroform-*d*) δ 72.2,

71.1, 70.9 (multiple peaks), 70.8, 63.6, 59.3, 41.5. HRMS (ES-ToF): 487.3115 [M+H$^+$] (calc. 487.3113).

2,5,8,11,14-Pentaoxaheptadecan-17-ol, 16-(2,5,8,11,14-pentaoxapentadec-1-yl)-, 17-(4-methyl benzenesulfonate) (S4)

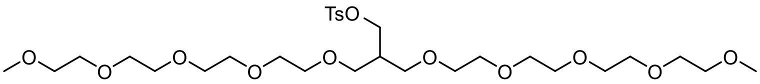

S3 (8.04 g, 16.5 mmol) was added dropwise to a solution of sodium hydroxide (3.96 g, 99.0 mmol) in a mixture of distilled water and tetrahydrofuran (1:1 v/v, 19.8 mL) at 0 °C. The reaction mixture was slowly treated with a solution of *p*-toluenesulfonyl chloride (4.09 g, 21.5 mmol) in THF (100 mL) and stirred for 2 h at 0 °C. The resulting suspension was stirred overnight at room temperature. The mixture was quenched with an aqueous HCl solution (2 M, 165 mL) and extracted with chloroform. The organic layers were washed with distilled water, dried over anhydrous $MgSO_4$ and concentrated *in vacuo*. Following column chromatography (ethyl acetate, then ethyl acetate: methanol, 90:10), the title product (4.15 g, 40%) was isolated as a pale yellow. ^{1}H NMR (400 MHz, chloroform-*d*) δ 7.78 (d, J = 8.3 Hz, 2H), 7.34 (d, J = 8.3 Hz, 2H), 4.11 (d, J = 5.5 Hz, 2H), 3.68 – 3.41 (m, 36H), 3.37 (s, 6H), 2.45 (s, 3H), 2.24 (hept, J = 5.5 Hz, 1H). ^{13}C NMR (100 MHz, chloroform-*d*) δ 145.0, 133.2, 130.1, 128.2, 72.1, 70.8 – 70.7 (multiple peaks), 70.6, 68.9, 68.6, 59.3, 39.8, 21.9. HRMS (ES-ToF): 641.3215 [M+H$^+$] (calc. 641.3207).

16-(Azidomethyl)-2,5,8,11,14,18,21,24,27,30-decaoxahentriacontane (S5)

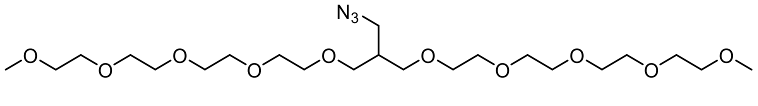

Sodium azide (0.24 g, 3.70 mmol) was added in one portion to a solution of **S4** (0.86 g, 1.37 mmol) in anhydrous DMF (14 mL). The mixture was stirred for 2 h at 100 °C and allowed to reach room temperature overnight. The mixture was diluted with ethyl acetate (100 mL) and

the resulting precipitate was removed by filtration. The solvent was removed *in vacuo* and the crude mixture was filtered through a silica plug (ethyl acetate), which afforded the title product (0.59 g, 84%) as a brown oil. ^{1}H NMR (400 MHz, chloroform-*d*) δ 3.71 – 3.44 (m, 38H), 3.38 (s, 6H), 2.16 (hept, J = 6.3 Hz, 1H). ^{13}C NMR (100 MHz, chloroform-*d*) δ 73.0, 71.0 (multiple peaks), 70.9, 70.8, 69.3, 59.4, 50.6, 42.4. HRMS (ES-ToF): 534.2990 [M+Na^{+}] (calc. 534.3003).

2-(2,5,8,11,14- Pentaoxapentadec -1-yl)-4,7,10,13,16-pentaoxaheptadecan-1-amine (S6)

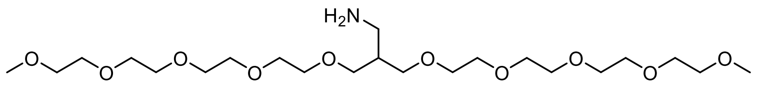

Triphenyl phosphine (0.68 g, 2.03 mmol) was added in small portions to a solution of **S5** (0.40 g, 0.78 mmol) in anhydrous THF (5.5 mL) at 0 °C. The mixture was allowed to reach room temperature and stirred for 6 h. The mixture was then heated to 50 °C for 1 h. After cooling to room temperature, the reaction mixture was quenched with distilled water (0.1 mL) and stirred overnight at room temperature. The solvent was removed *in vacuo* and the crude residue was suspended in distilled water (35 mL). The aqueous portion was thoroughly washed with diethyl ether and concentrated under reduced pressure to give the title product (0.31 g, 82%) as a pale yellow oil. The crude product was used in the next step without further purification. ^{1}H NMR (400 MHz, chloroform-*d*) δ 3.68 – 3.49 (m, 36H), 3.38 (s, 6H), 2.79 (d, J = 6.0 Hz, 2H), 2.25 (hept, J = 6.0 Hz, 1H). ^{3}C NMR (100 MHz, chloroform-*d*) δ 72.3, 71.2, 70.9 (multiple peaks), 70.8, 59.4, 42.5, 42.3. HRMS (ES-ToF): 486.3278 [M+H^{+}] (calc. 486.6230).

Synthesis of 4gNDTI-Br

2,6-Dibromo-1,4,5,8-tetracarboxynaphthalenediimide (1)

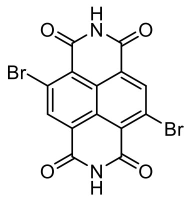

Following the procedure reported by Dawson et al.,[1] a suspension of 2,6-dibromonaphthalene-1,4,5,8-tetracarboxydianhydride (5.0 g, 11.7 mmol) and ammonium acetate (18.1 g, 235 mmol) in glacial acetic acid (100 mL) was heated under reflux for 2 h. After cooling to room temperature, the precipitate was collected by filtration and washed with acetic acid and methanol to afford the title product (4.79 g, 97%) as an orange solid: ^{1}H NMR (400 MHz, DMSO-d_6) δ 12.28 (br s, 2H), 8.65 (s, 2H); HRMS (ES-ToF): 420.8465 [M-H^{+}] (calc. 420.8640).

Tributyl(triethylsilylethynyl)tin (2)

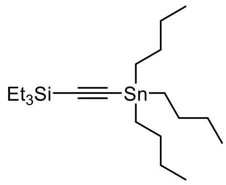

n-BuLi (2.5 M in hexane, 10.3 mL, 25.8 mmol) was added dropwise to a solution of triethylsilylacetylene (4.4 mL, 24.6 mmol) in anhydrous THF (100 mL) at –78 °C. The reaction mixture was stirred for 1 h at –78 °C. Tributyltinchloride (6.7 mL, 24.6 mmol) was added dropwise and the resulting mixture was allowed to reach room temperature overnight. The mixture was quenched with a saturated aqueous NH_4Cl solution (20 mL) and extracted with hexane (3 × 200 mL). The combined organic layers were washed with distilled water, brine and dried over anhydrous Na_2SO_4. Removal of the solvent *in vacuo* afforded the crude product (9.97 g, 94%) as a colourless oil. The crude product was used in the next step without further

purification. ^{1}H NMR (400 MHz, chloroform-*d*) δ 1.65 – 1.49 (m, 6H), 1.38 – 1.29 (m, 6H), 1.09 – 0.91 (m, 15H), 0.89 (t, J = 7.3 Hz, 9H), 0.58 (q, J = 7.3 Hz, 6H). ^{13}C NMR (100 MHz, chloroform-*d*) δ 117.1, 114.3, 28.9, 26.9, 14.4, 11.2, 7.5, 4.6.

2,6-Bis(triethylsilylethynyl)-1,4,5,8-naphthalene tetracarboxylic acid diimide (3)

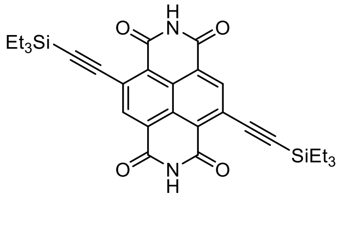

Bis(triphenylphosphine)palladium (II) dichloride (0.31 g, 0.45 mmol) was added to a solution of **2** (8.46 g, 19.71 mmol) and **1** (3.80 g, 8.96 mmol) in anhydrous, degassed toluene (120 mL). The mixture was stirred overnight at 120 °C under an argon atmosphere and after cooling, the solvent was removed *in vacuo*. Recrystallisation from tetrahydrofuran-methanol afforded the title compound (4.54 g, 93%) as a yellow solid: ^{1}H NMR (400 MHz, DMSO-d_6) δ 12.12 (br s, 2H), 8.40 (s, 2H), 1.09 (t, J = 7.9 Hz, 18H), 0.74 (q, J = 7.9 Hz, 12H). HRMS (ES-ToF): 541.1987 [M-H$^+$] (calc. 541.1979).

2,7-Bis(triethylsilyl)naphtho[2,3-b:6,7-b']dithiophene-4,5,9,10-tetracarboxylic acid diimide (4)

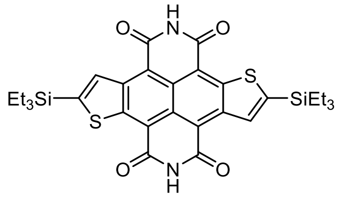

Modifying the procedure of Nakano et al.,[2] sodium sulfide nonahydrate (3.72 g, 15.49 mmol) was added in small portions to a suspension of **3** (1.40 g, 2.58 mmol) in anhydrous, degassed 2-methoxyethanol (250 mL) and acetic acid (8.7 mL) at 0 °C. The suspension was allowed to reach room temperature and stirred overnight under nitrogen. The mixture was then exposed to atmospheric conditions and compressed air was continuously bubbled into the

stirred reaction mixture for 72 h at room temperature. A gradual colour change from brown to maroon was observed. The reaction mixture was cooled to 0 °C and poured over distilled water (200 mL). The resulting precipitate was collected under vacuum and thoroughly washed with distilled water and methanol. The solid was suspended in hot chloroform (60 mL) and the insoluble residues were removed by filtration. The filtrate was concentrated under reduced pressure and the resulting solid was recrystallised two times from chloroform-methanol to give the title product (0.35 g, 22%) as a maroon solid. ^{1}H NMR (400 MHz, chloroform-*d*) δ 9.10 (s, 2H), 9.00 (s, 2H), 1.13 – 1.03 (m, 30H). ^{13}C NMR (100 MHz, chloroform-*d*) δ 163.3, 163.2, 158.2, 148.6, 143.7, 131.2, 124.5, 118.8, 116.9, 7.8, 4.4. HRMS (ES-ToF): 606.1509 (calc. 606.1499).

4gNDTI-TES

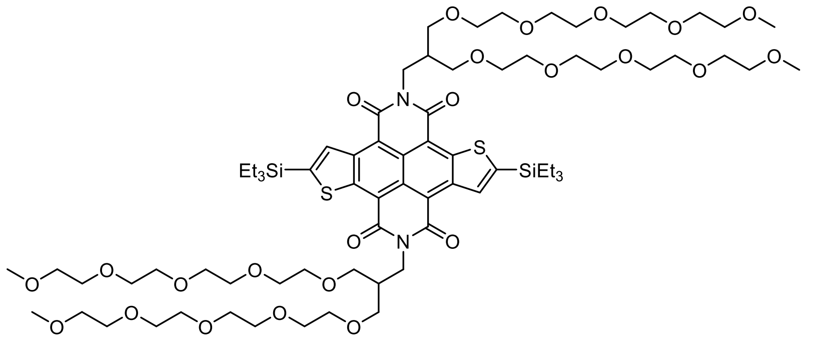

Diethylazodicarboxylate (40 wt. % in toluene, 0.89 mL, 1.96 mmol) was added dropwise to a solution of **4** (300 mg, 0.49 mmol), **S3** (600 mg, 1.23 mmol) and triphenylphosphine (0.51 g, 1.96 mmol) in degassed anhydrous THF (12 mL) at 0 °C. The solution was allowed to reach room temperature and stirred overnight. The solvent was removed under reduced pressure and the crude residues were subjected to column chromatography (ethyl acetate, then ethyl acetate: methanol, 90:10) and preparative gel permeation chromatography in chloroform to give the title product (690 mg, 90%) as a dark red viscous oil. ^{1}H NMR (400 MHz, chloroform-*d*) δ 9.15 (s, 2H), 4.48 (d, J = 6.7 Hz, 4H), 3.65 – 3.45 (m, 72H), 3.34 (s, 12H), 2.69 (hept, J = 6.7 Hz, 2H), 1.18 – 0.94 (m, 30H). ^{13}C NMR (100 MHz, chloroform-*d*) δ 164.1, 163.9, 156.9,

148.6, 144.3, 131.7, 123.0, 118.8, 117.6, 72.2, 71.3, 70.9 – 70.8 (multiple peaks), 70.7, 59.3, 41.8, 38.8, 7.8, 4.4. HRMS (ES-ToF): 1543.7468 [M+H$^+$] (calc. 1543.7446).

4gNDTI-Br

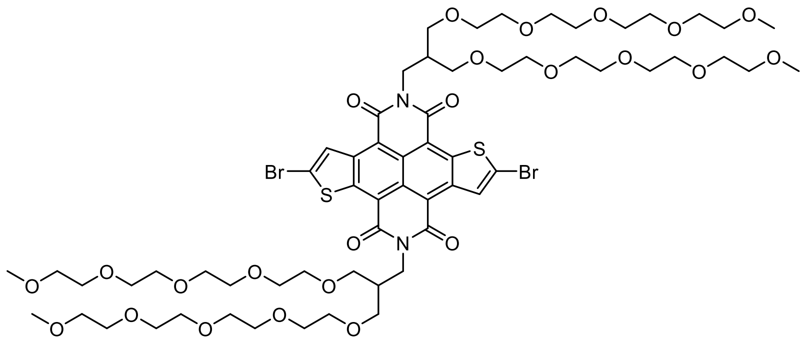

Bromine (0.06 mL, 1.25 mmol) was added to a solution of **4gNDTI-TES** (380 mg, 0.25 mmol) in degassed anhydrous DCM (10 mL) at 0 °C. The resulting solution was heated to 40 °C and stirred for 3 h. After cooling to room temperature, the solvent was evaporated and the crude residues were subjected to column chromatography (ethyl acetate, then ethyl acetate: methanol, 90:10) and preparative gel permeating chromatography in chloroform to afford the title product (185 mg, 51%) as a dark red viscous oil. ^{1}H NMR (400 MHz, chloroform-*d*) δ 9.05 (s, 2H), 4.42 (d, J = 6.6 Hz, 4H), 3.70 – 3.40 (m, 72H), 3.35 (s, 12H), 2.60 (hept, J = 6.6 Hz, 2H). ^{13}C NMR (100 MHz, chloroform-*d*) δ 163.3, 163.0, 146.2, 142.3, 133.3, 127.0, 122.1, 117.8, 116.2, 72.2, 71.2, 70.8 – 70.6 (multiple peaks), 59.3, 41.8, 38.7. HRMS (ES-ToF): 1473.3893 [M+H$^+$] (calc. 1473.3900).

Synthesis of 4gNDI-Br

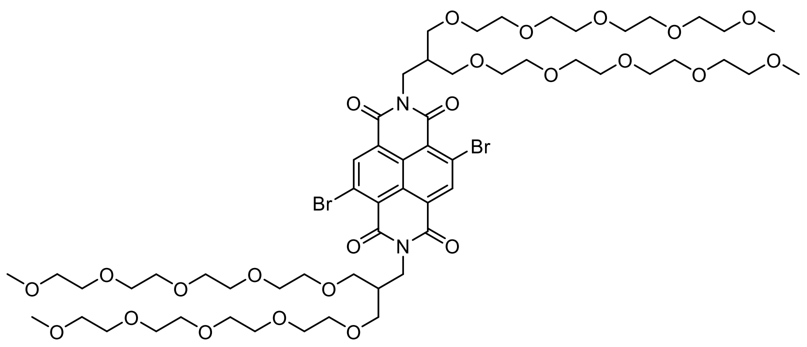

A suspension of 2,6-dibromonaphthalene-1,4,5,8-tetracarboxydianhydride (0.10 g, 0.23 mmol) and anhydrous zinc acetate (84.4 mg, 0.46 mmol) in anhydrous *o*-xylene (7 mL) was heated to 80 °C, after which **S6** (0.25 g, 0.51 mmol) was added dropwise. After heating to 130 °C for 2 h, the reaction mixture was diluted with chloroform and washed with water. The aqueous layer was further extracted with chloroform and the combined organic portions were washed with water and dried over anhydrous $MgSO_4$. The solvent was removed *in vacuo* and the resulting red residues were purified by column chromatography (ethyl acetate, then ethyl acetate: methanol, 90:10) and preparative gel permeation chromatography in chloroform, which gave the title product (85 mg, 27%) as a waxy yellow solid. ^{1}H NMR (400 MHz, chloroform-*d*) δ 8.97 (s, 2H), 4.32 (d, J = 6.2 Hz, 4H), 3.66 – 3.46 (m, 72H), 3.37 (s, 12H), 2.52 (hept, J = 6.2 Hz, 2H). ^{13}C NMR (100 MHz, chloroform-*d*) δ 161.4, 161.3, 139.3, 128.5, 128.0, 125.7, 124.4, 72.2, 71.3, 70.9 – 70.8 (multiple peaks), 70.7, 59.4, 42.2, 38.6. HRMS (ES-ToF): 1361.4431 (calc. 1361.4459).

Synthesis of the polymers

P4gNDTI

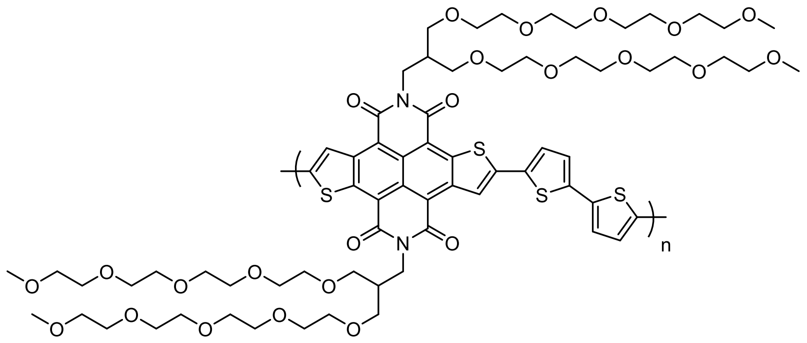

A mixture of **4gNDTI-Br** (71.75 mg, 48.70 µmol 1.0 equiv), 5,5'-bis(trimethylstannyl)-2,2'-bithiophene (23.95 mg, 48.70 µmol, 1.0 equiv) , $Pd_2(dba)_3$ (0.89 mg, 0.97 µmol, 0.02 equiv) and P(*o*-tol)$_3$ (1.18 mg, 3.89 µmol, 0.08 equiv) in anhydrous, degassed chlorobenzene (2.0 mL) was degassed for 10 min and heated to 135 °C overnight. 0.1 mL of a solution of 2-(tributylstannyl)thiophene (0.1 mL) and $Pd_2(dba)_3$ (1.00 mg, 1.09 µmol) in anhydrous, degassed chlorobenzene (0.5 mL) were added and the resulting mixture was stirred for 1 h at 135 °C. 0.1 mL of a solution of 2-bromothiophene (0.1 mL) in anhydrous, degassed chlorobenzene (0.5 mL) were subsequently added and stirring at 135 °C was maintained for a further 1 h. The reaction mixture was cooled to room temperature and precipitated in ethyl acetate, followed by the addition of hexane. The solid was collected in a thimble and Soxhlet extraction was carried out with hexane, methanol, ethyl acetate, acetone, THF and chloroform. The polymer was dissolved in hot chloroform and re-precipitated in ethyl acetate, followed by the addition of hexane. Polymer **P4gNDTI** was obtained as a black green solid is a yield of 58% (42 mg, 28.42 µmol). ^{1}H NMR (400 MHz, chloroform-*d*) δ 9.16 – 8.55 (broad), 4.57 – 4.53 (br s), 3.92 – 3.31 (m), 2.78 – 2.51 (br s). GPC (chloroform, 40 °C): M_n = 59.4 kDa, M_w = 269.4 kDa.

P4gNDI

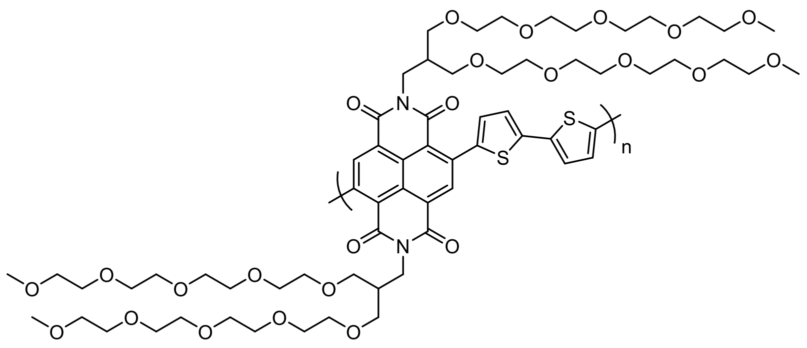

A mixture of **4gNDI-Br** (49.62 mg, 36.45 µmol, 1.0 equiv), 5,5'-bis(trimethylstannyl)-2,2'-bithiophene (17.93 mg, 36.45 µmol, 1.0 equiv) , $Pd_2(dba)_3$ (0.67 mg, 0.73 µmol, 0.02 equiv) and P(*o*-tol)$_3$ (0.89 mg, 2.92 µmol, 0.08 equiv) in anhydrous, degassed chlorobenzene (1.5 mL) was degassed for 10 min and heated to 135 °C overnight. 0.1 mL of a solution of 2-(tributylstannyl)thiophene (0.1 mL) and $Pd_2(dba)_3$ (1.00 mg, 1.09 µmol) in anhydrous, degassed chlorobenzene (0.5 mL) were added and the resulting mixture was stirred for 1 h at 135 °C. 0.1 mL of a solution of 2-bromothiophene (0.1 mL) in anhydrous, degassed chlorobenzene (0.5 mL) were subsequently added and stirring at 135 °C was maintained for a further 1 h. The reaction mixture was cooled to room temperature and precipitated in a mixture of ethyl acetate: hexane (1:1, v/v). The solid was collected in a thimble and Soxhlet extraction was carried out with hexane and ethyl acetate. The polymer was dissolved in hot ethyl acetate and re-precipitated in ethyl acetate: hexane (1:1, v/v). Polymer **P4gNDI** was obtained as a deep blue solid is a yield of 48% (24 mg, 17.57 µmol). ^{1}H NMR (400 MHz, chloroform-*d*) δ 8.82 (br s, 2H), 7.40 (br s, 4H), 4.29 (br s, 4H), 3.66 – 3.47 (m, 72H), 3.35 (s, 12H), 2.56 (br s, 2H). GPC (chloroform, 40 °C): M_n = 22.0 kDa, M_w = 36.0 kDa.

GPC measurements

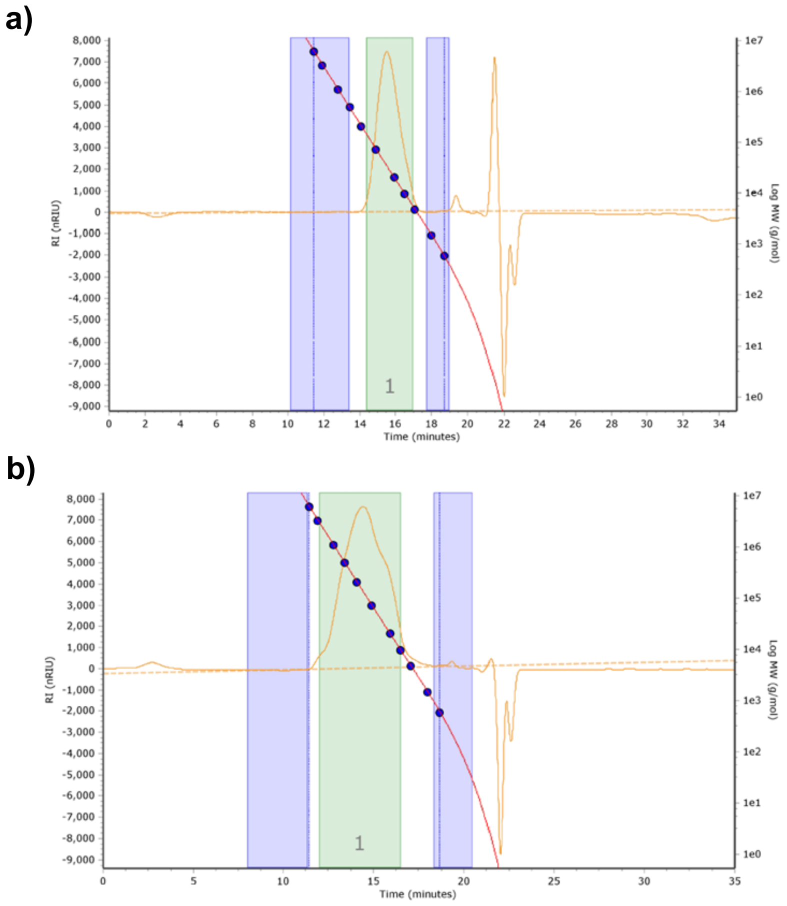

Figure S1. GPC traces of (a) **P4gNDI** (b) **P4gNDTI** in chloroform at 40 °C calibrated using low dispersity (<1.10) polystyrene standards. The bimodal distribution observed for **P4gNDTI** is most likely a result of aggregate formation in solution.

Thermogravimetric analysis

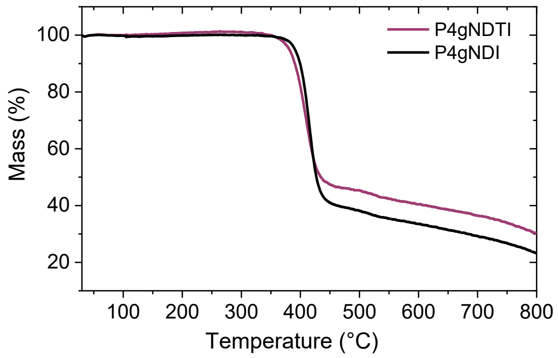

Figure S2. Thermogravimetric analysis of the polymers. Polymers were heated under inert conditions (nitrogen flow) under the following conditions: [1] 30.0 to 105.0 °C, 34.99 K min^{-1}, N_2 50.0 mL min^{-1}, [2] 105.0 °C, 10.00 min, N_2 50.0 mL min^{-1} [3] 105.0 to 800.0 °C, 10.00 K min^{-1}, N_2 50.0 mL min^{-1}.

DFT calculations

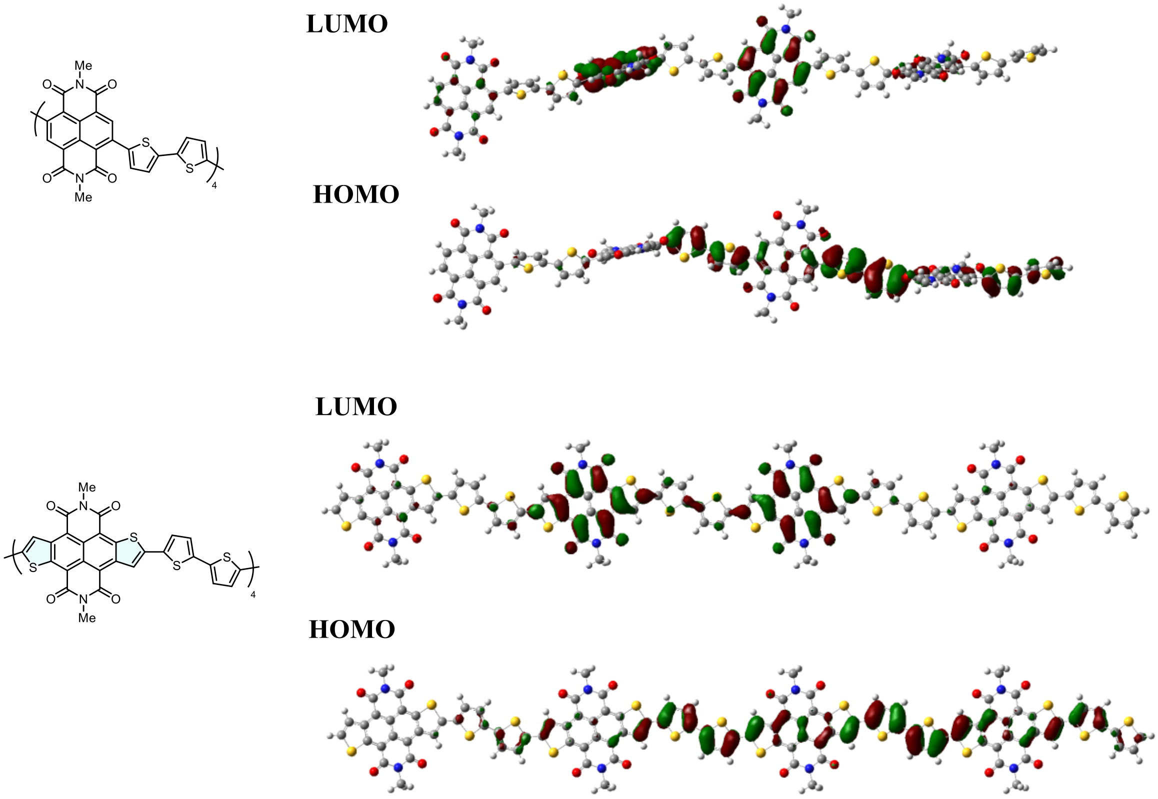

Figure S3. DFT calculations of methyl-substituted NDI-T2 and NDTI-T2 tetramers at the wB97XD/6-31G* level of theory showing the energy-minimized structures and HOMO and LUMO wave function distributions.

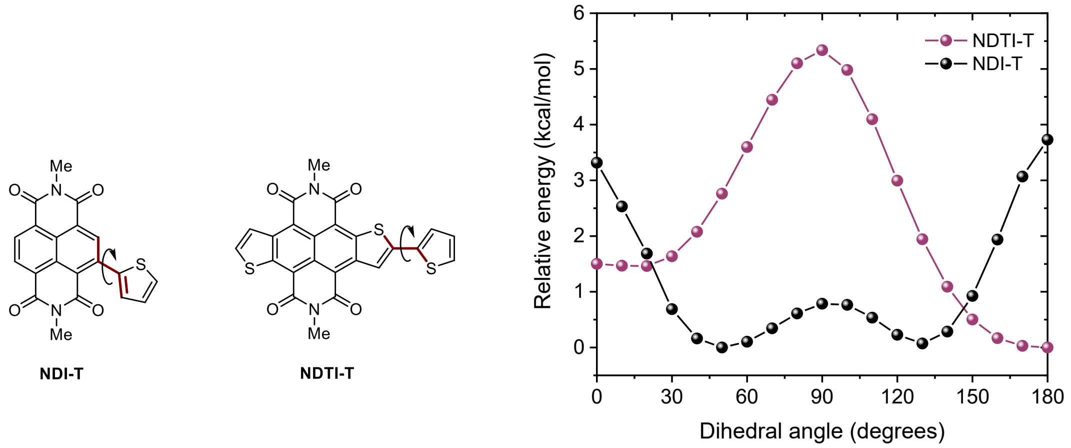

Figure S4. Torsional energy profile of the NDI-T and NDTI-T units calculated using B3LYP-D3/6-31G* in gas phase.

UV-Vis measurements

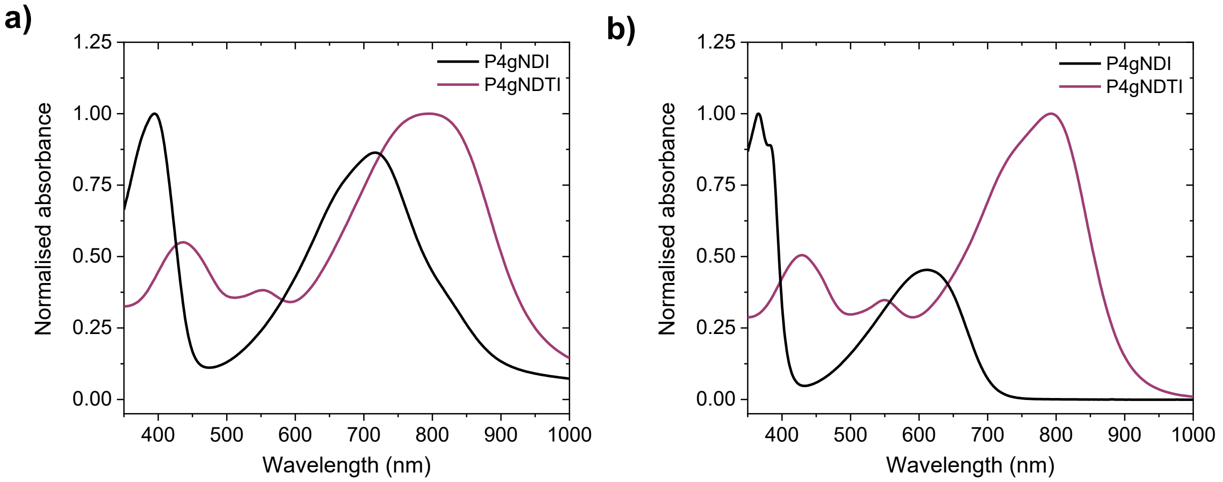

Figure S5. Normalized UV-Vis spectra of polymer (a) thin films on glass substrate and (b) in chloroform solution at room temperature.

GIWAXS analysis

Table S1. GIWAXS peak parameters

d-spacings (Å)	In-plane					Out-of-plane
	(100)	(001)	(001)'	(002)'	(010)	(010)
P4gNDI	28.4	14.5	7.41	7.43	--	4.09
P4gNDTI	28.4	--	--	--	3.62	3.67

Cyclic voltammetry in organic electrolytes

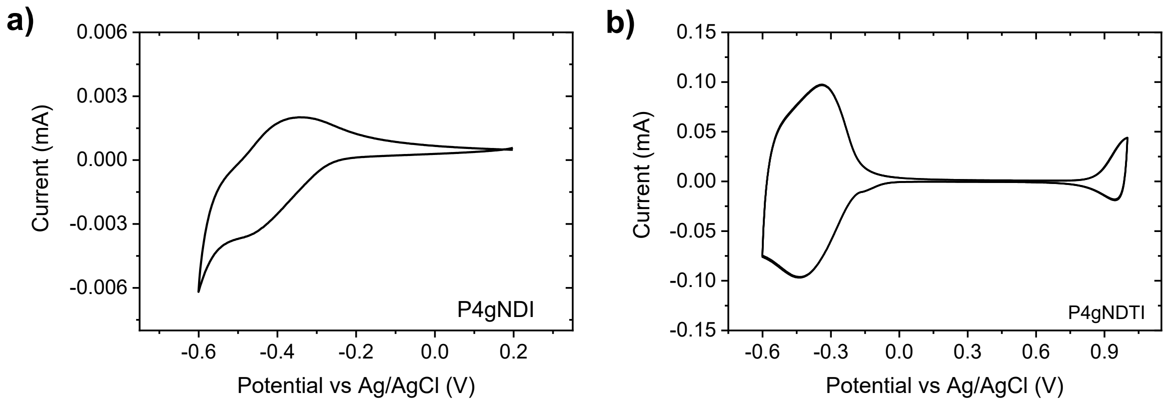

Figure S6. Cyclic voltammetry of (a) **P4gNDI** (b) **P4gNDTI** on glassy carbon electrodes in acetonitrile using TBAPF6 as the supporting electrolyte (0.1 M) at 100 mV s^{-1}, showing 2nd, 3rd and 4th cycles for **P4gNDTI**, and 2nd cycle only for **P4gNDI** as the polymer film is not stable in contact with the organic electrolyte.

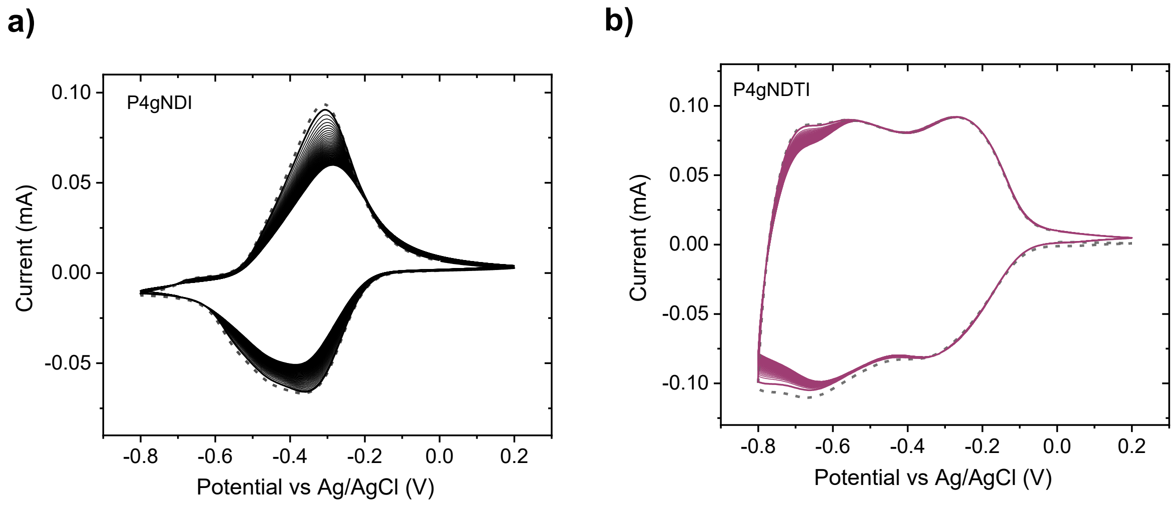

Figure S7. Cycling stability (50 scans) of (a) **P4gNDI** and (b) **P4gNDTI** films on glassy carbon electrodes in degassed 0.1 M NaCl aqueous solution with a scan rate of 100 mV s^{-1} and a Ag/AgCl reference electrode. The dashed lines show the first cycle.

Spectroelectrochemistry measurements

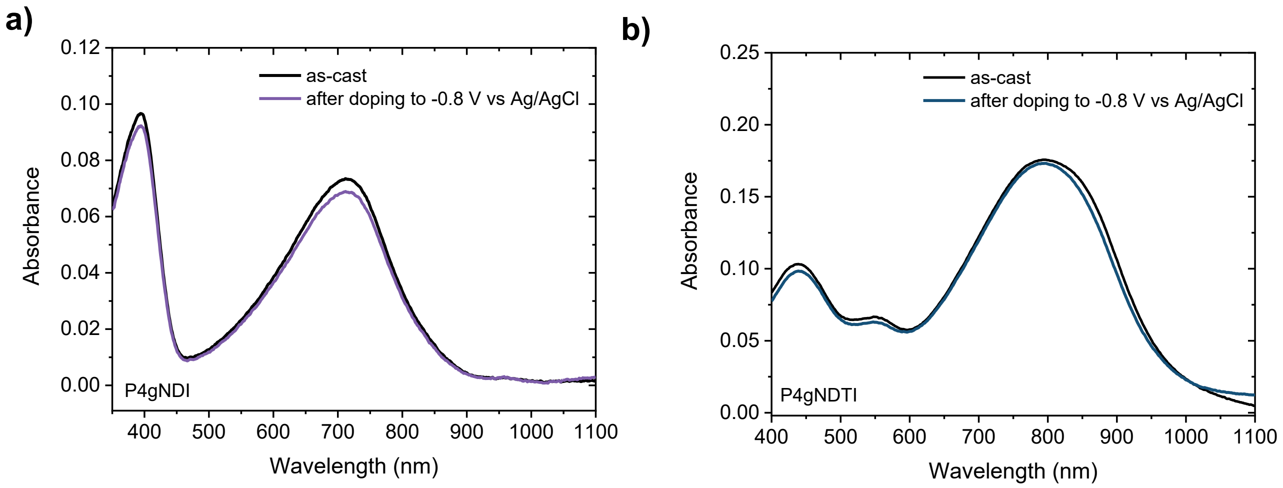

Figure S8. UV-Vis Spectra of (a) **P4gNDI** and (b) **P4gNDTI** prior to and after charging to –0.8 V versus Ag/AgCl in 0.1 M NaCl aqueous solution.

QCM-D measurements

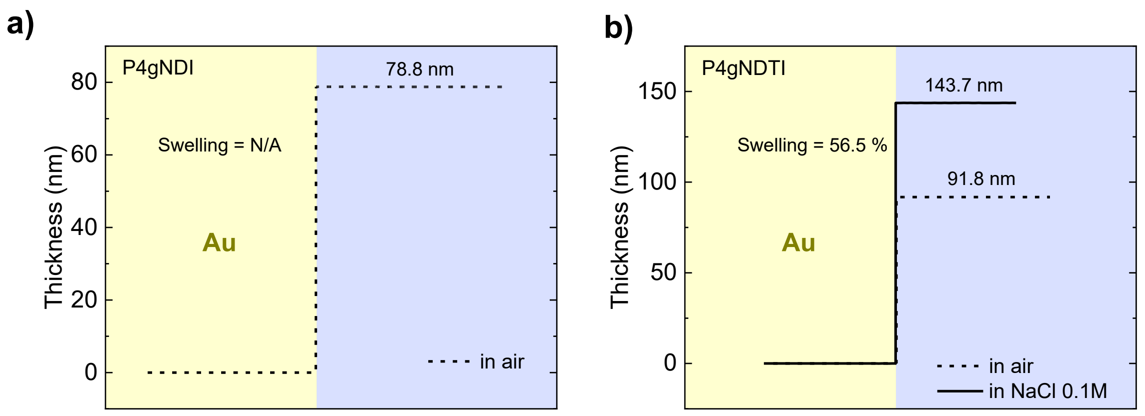

Figure S9. QCM-D measurements of the bare Au sensors before coating (yellow) and after coating with the polymer films (blue), showing the thickness of a) **P4gNDI** and b) **P4gNDTI** films in air and in a 0.1 M NaCl aqueous solution.

EQCM-D measurements

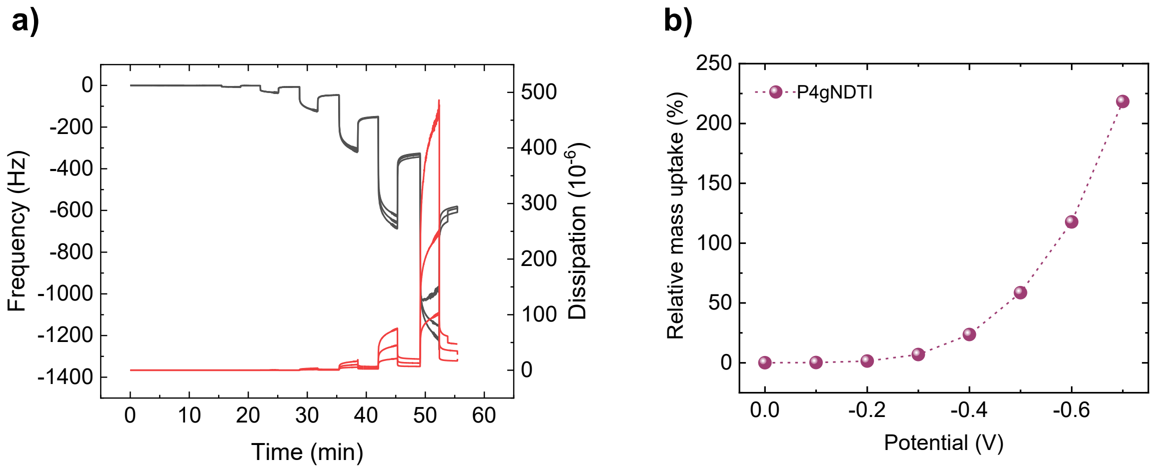

Figure S10. a) 3rd, 5th and 7th frequency (gray) and dissipation (red) overtones vs time with increasing doping potential (0 to –0.7 V vs Ag/AgCl) and b) swelling percentage from eQCM-D measurements with applied potentials between 0 and –0.7 V versus Ag/AgCl in 0.1 M NaCl aqueous solution for **P4gNDTI**.

OECT measurements

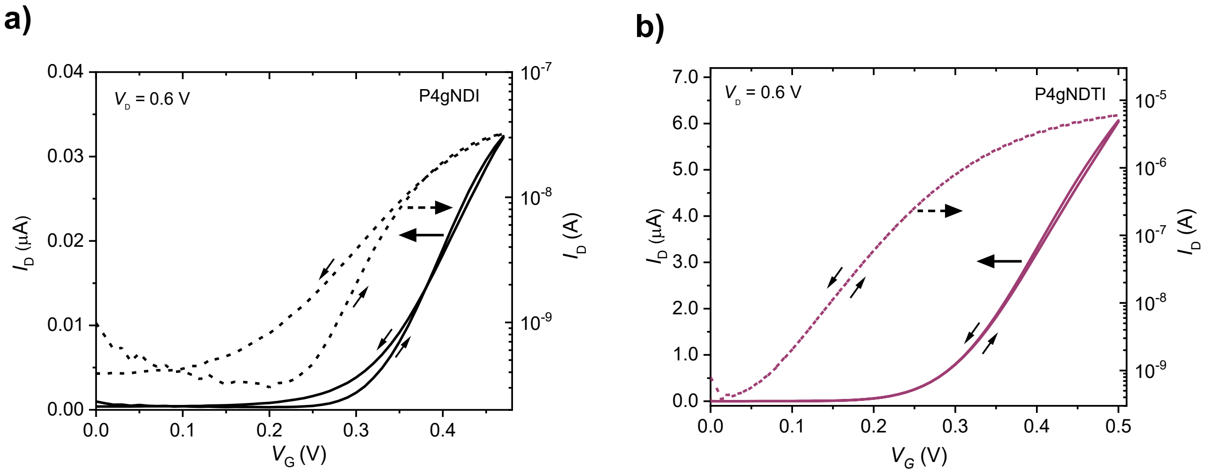

Figure S11. Performance of a) **P4gNDI** (d = 1.26 ± 0.10 µm) and b) **P4gNDTI** (d = 0.59 ± 0.12 µm) OECT devices (W = 100 µm, L = 10 µm, 200 mV/s): transfer curves at V_D = 0.6 V on a linear and logarithmic scale (dotted lines), showing hysteresis with direction indicated with arrows. All measurements were performed in ambient conditions in 0.1 M NaCl with a Ag/AgCl pellet gate electrode.

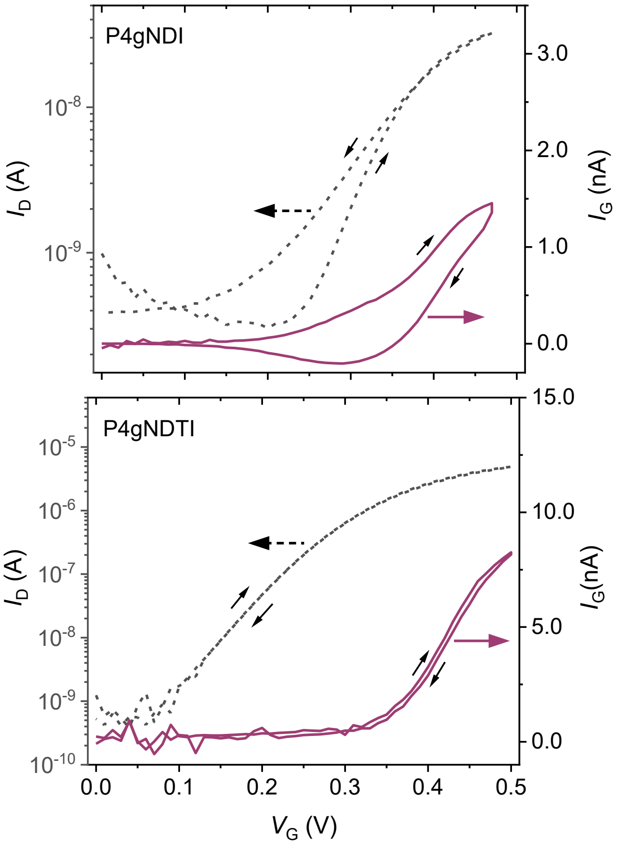

Figure S12. Saturation regime transfer curves with measured gate current of **P4gNDI** (top) and **P4gNDTI** (bottom) OECTs at $V_D = 0.6$ V. All measurements were performed in ambient conditions in 0.1 M NaCl with a Ag/AgCl pellet gate electrode.

EIS measurements

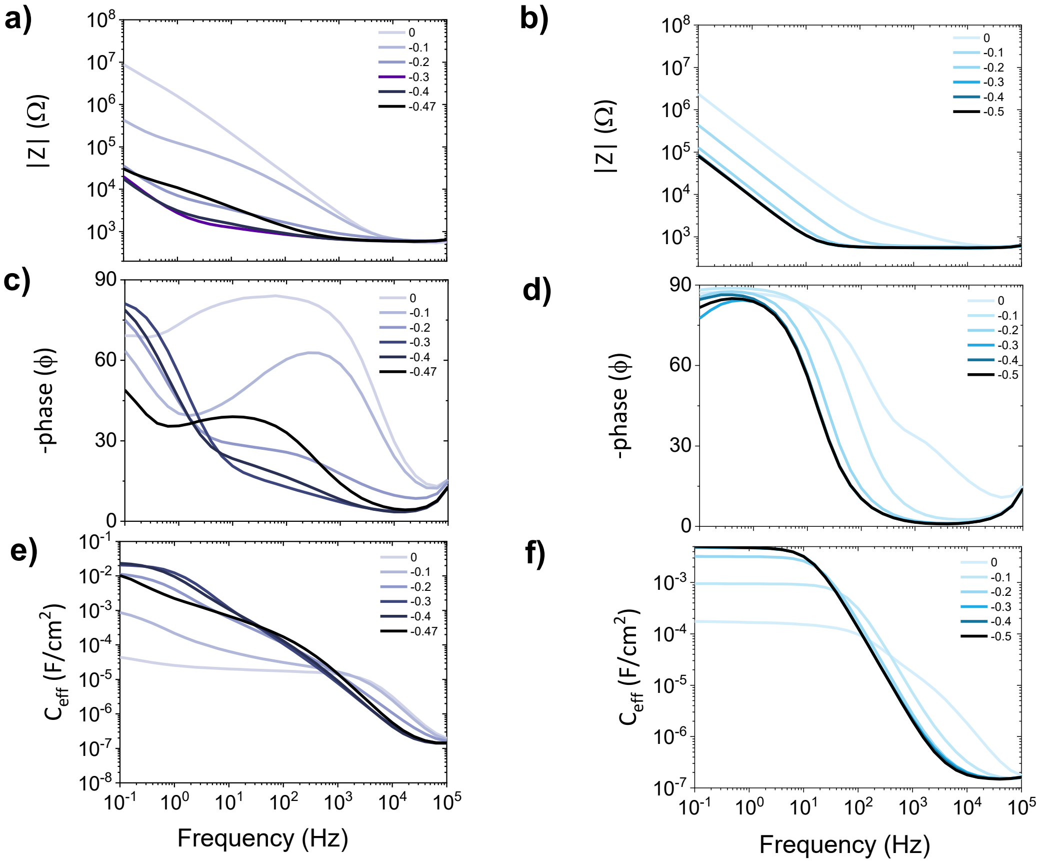

Figure S13. Electrochemical impedance spectroscopy of **P4gNDI** (left) and **P4gNDTI** (right) coated on micronscale Au patterns (600 × 600 µm) recorded in 0.1 M NaCl aqueous solution showing the a)-b) impedance magnitude, c)-d) the phase and e)-f) the corresponding capacitance versus frequency plots.

Stability of the OECT devices

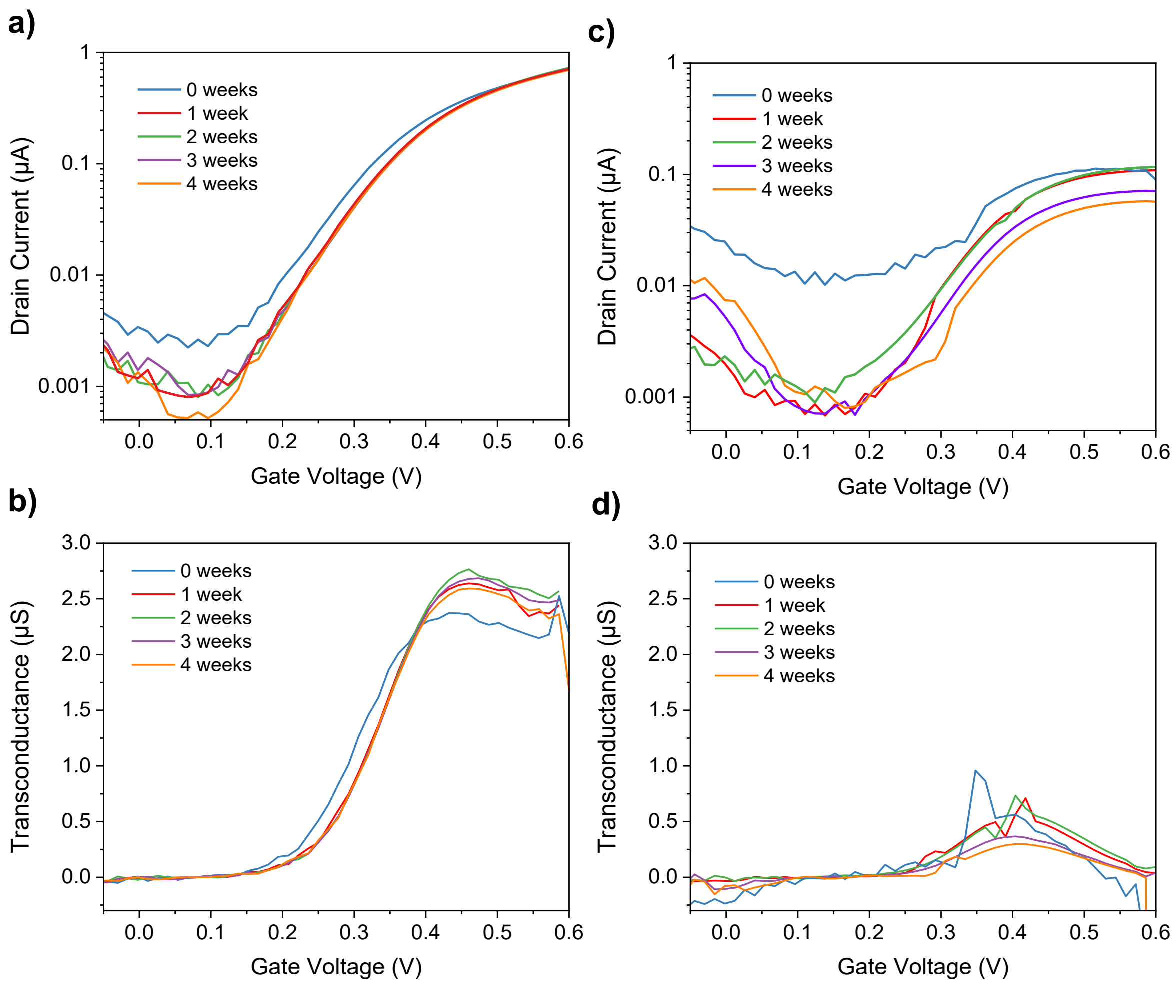

Figure S14. Ageing studies comparing **P4gNDTI** and **P4gNDI** in OECTs. (a) Transfer characteristics and (b) corresponding transconductance plots for **P4gNDTI** OECTs aged over 4 weeks. (c) Transfer characteristics and (d) corresponding transconductance plots for **P4gNDI** OECTs aged over 4 weeks. All data shown is for the transistor backwards sweep. Thin-film thicknesses were taken by averaging over 5 measurements within the OECT channel and were found to be 65 ± 3 and 50 ± 4 nm, for **P4gNDTI** and **P4gNDI** OECTs, respectively. The transistors have a channel length of 30 µm and a width of 600 µm, respectively, and are measured by applying a drain voltage of 0.6 V, whilst exposing to a 0.1 M NaCl electrolyte.

NMR Spectra

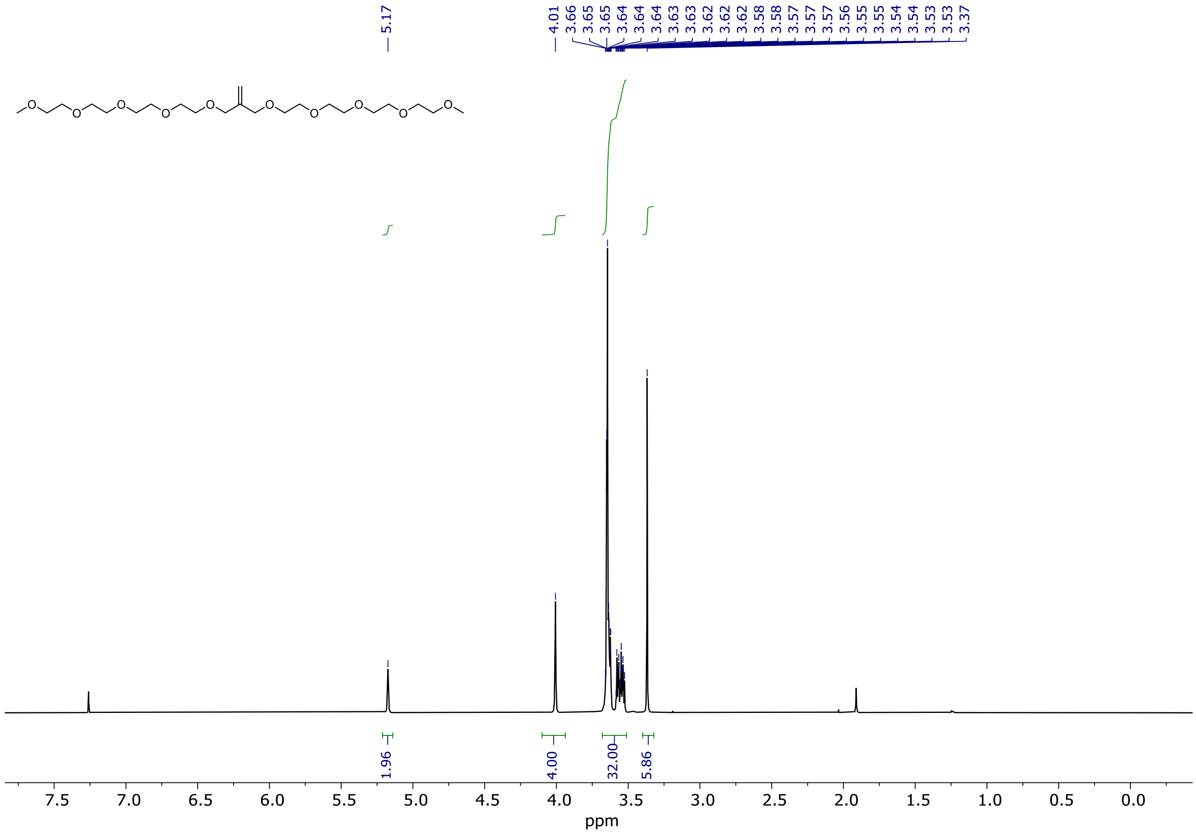

Figure S15. ^{1}H NMR spectra of **S2** in chloroform-*d*.

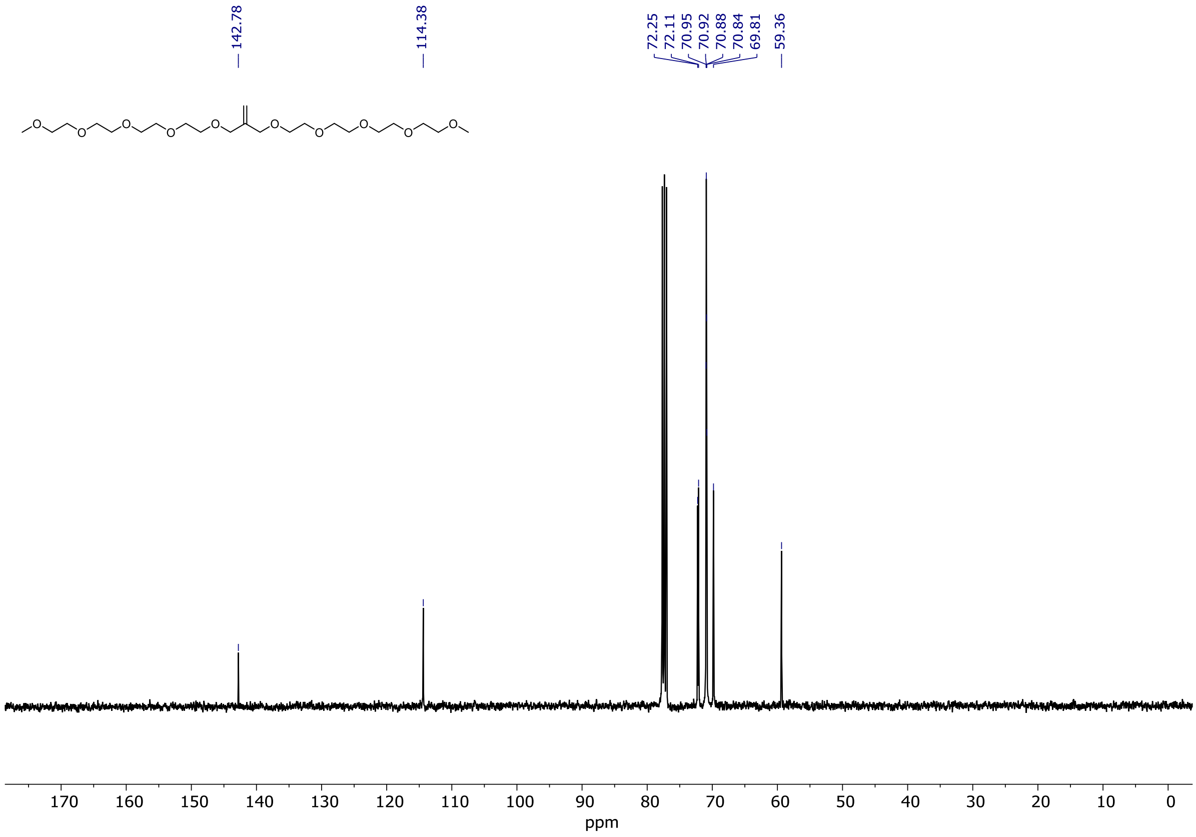

Figure S16. ^{13}C NMR spectra of **S2** in chloroform-*d*.

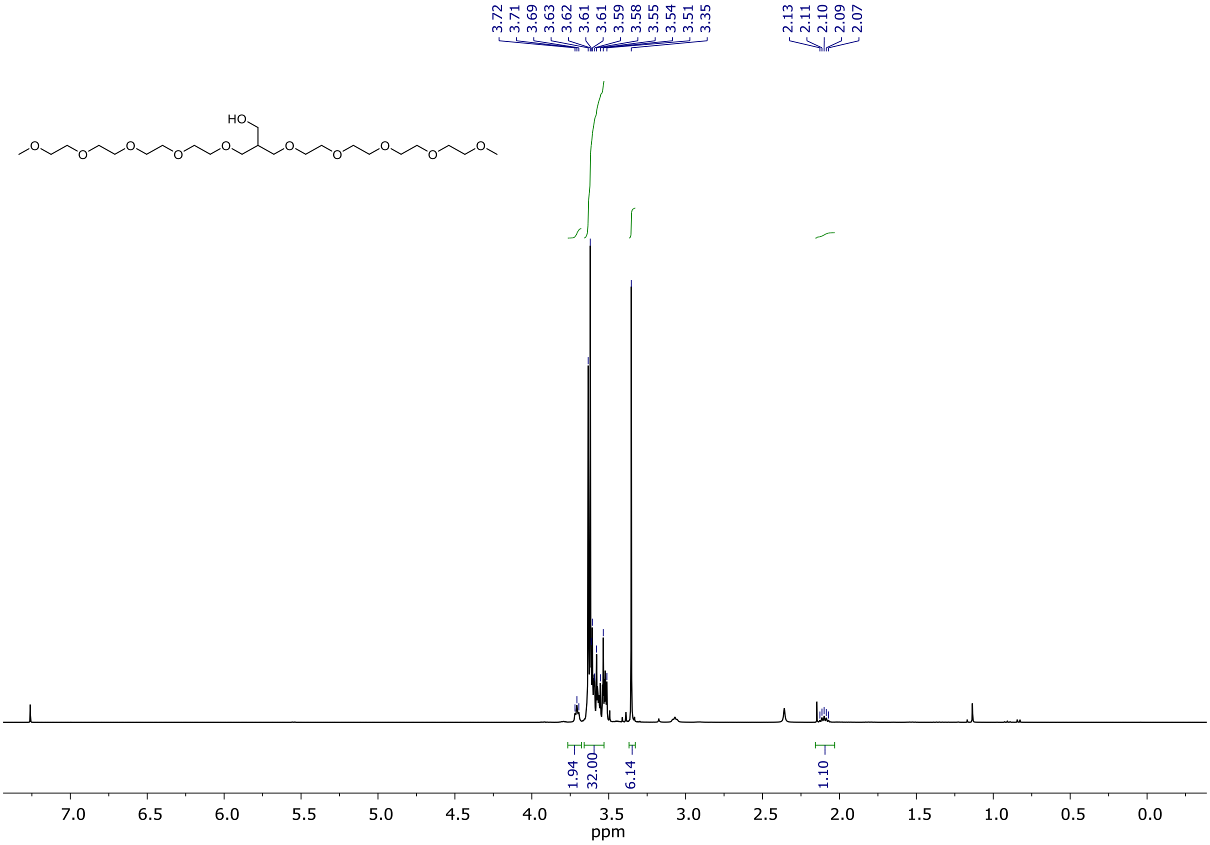

Figure S17. ^{1}H NMR spectra of **S3** in chloroform-*d*.

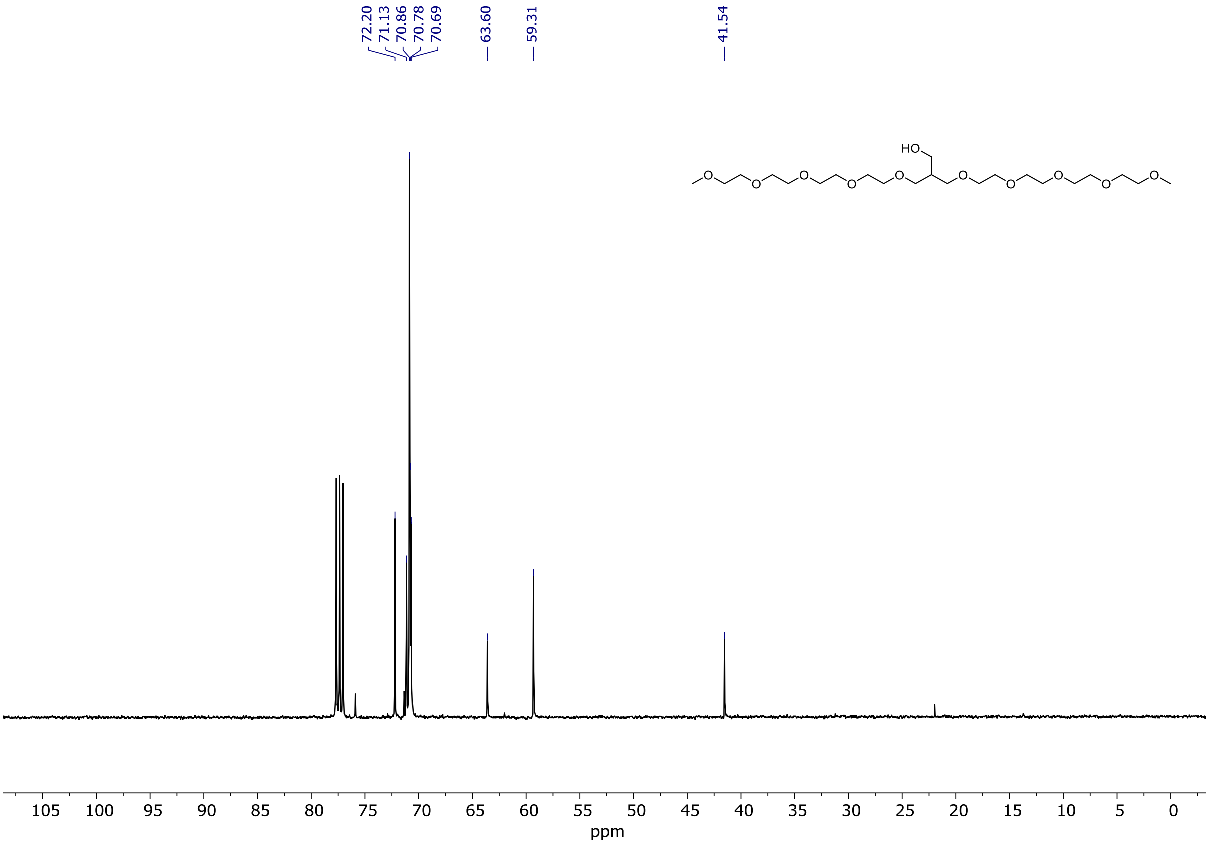

Figure S18. ^{13}C NMR spectra of **S3** in chloroform-*d*.

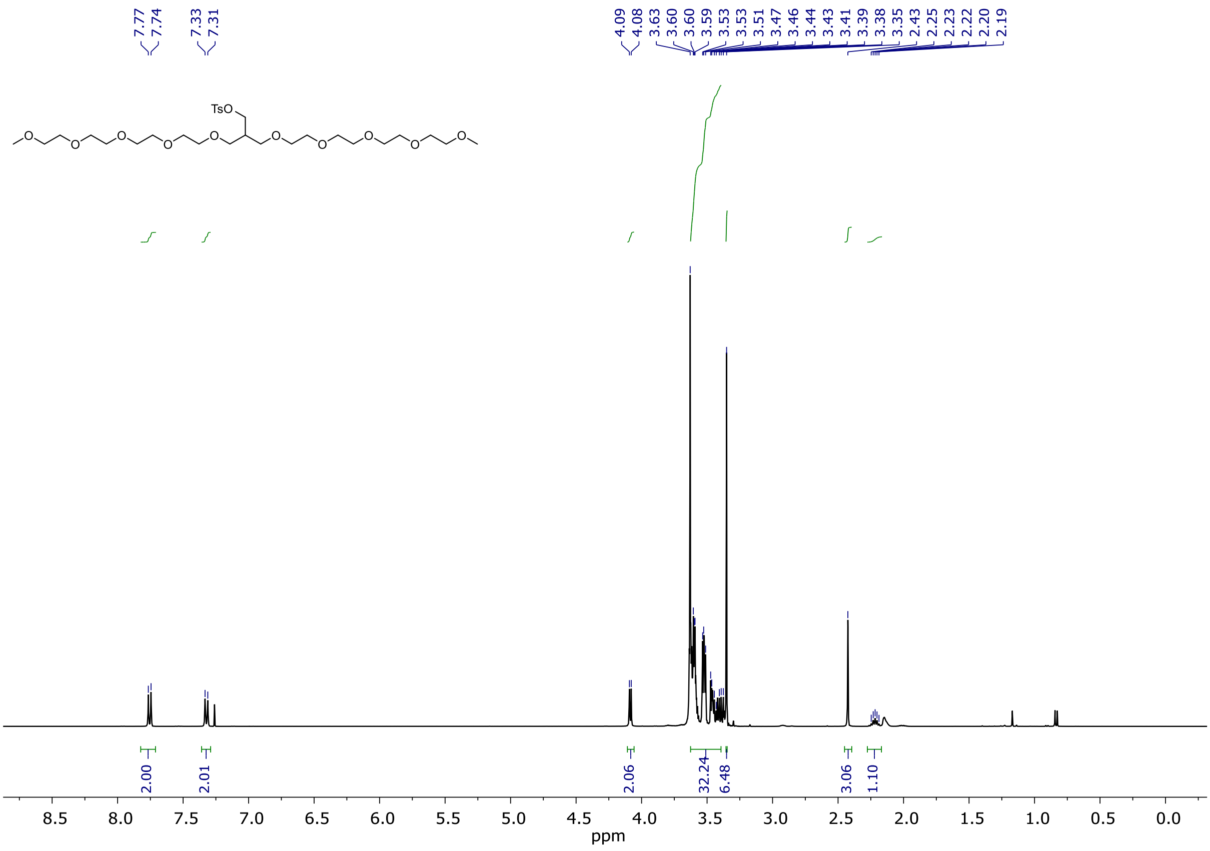

Figure S19. ^{1}H NMR spectra of **S4** in chloroform-*d*.

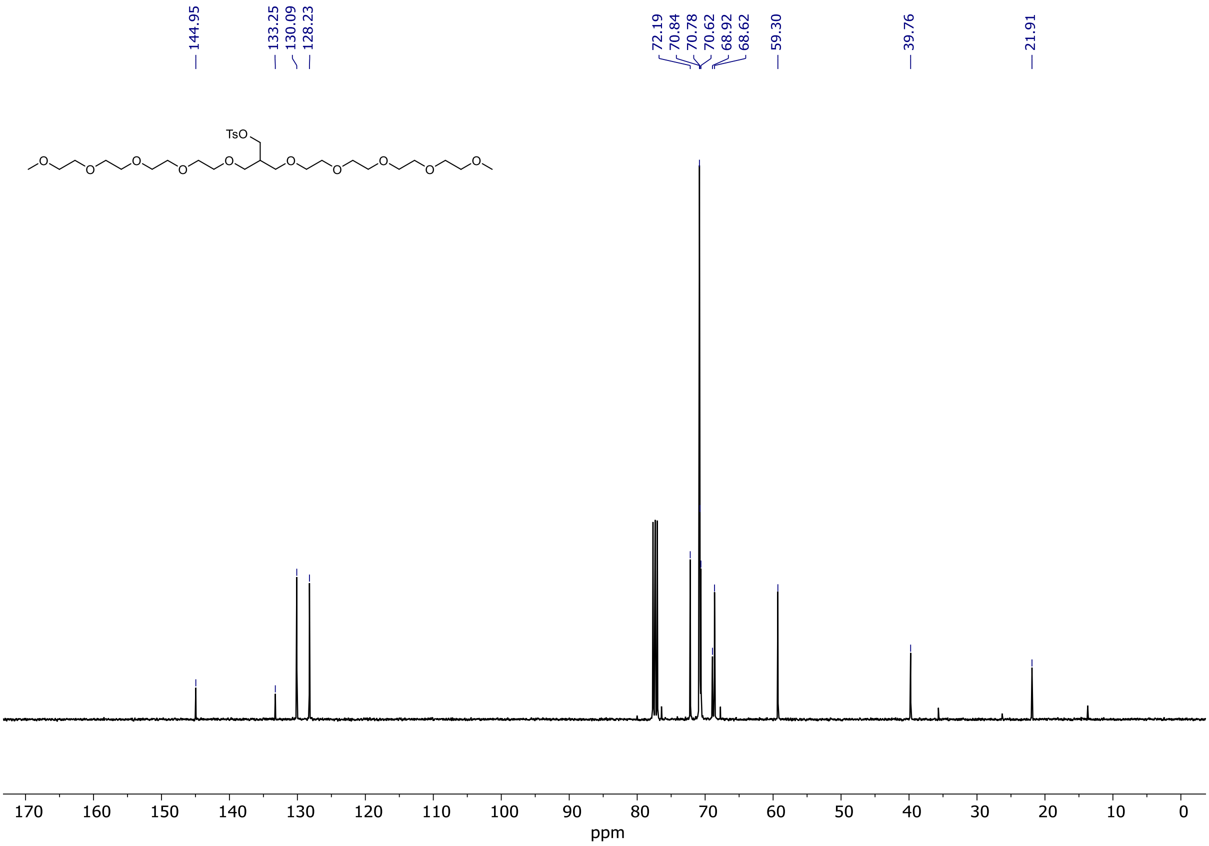

Figure S20. ^{13}C NMR spectra of **S4** in chloroform-*d*.

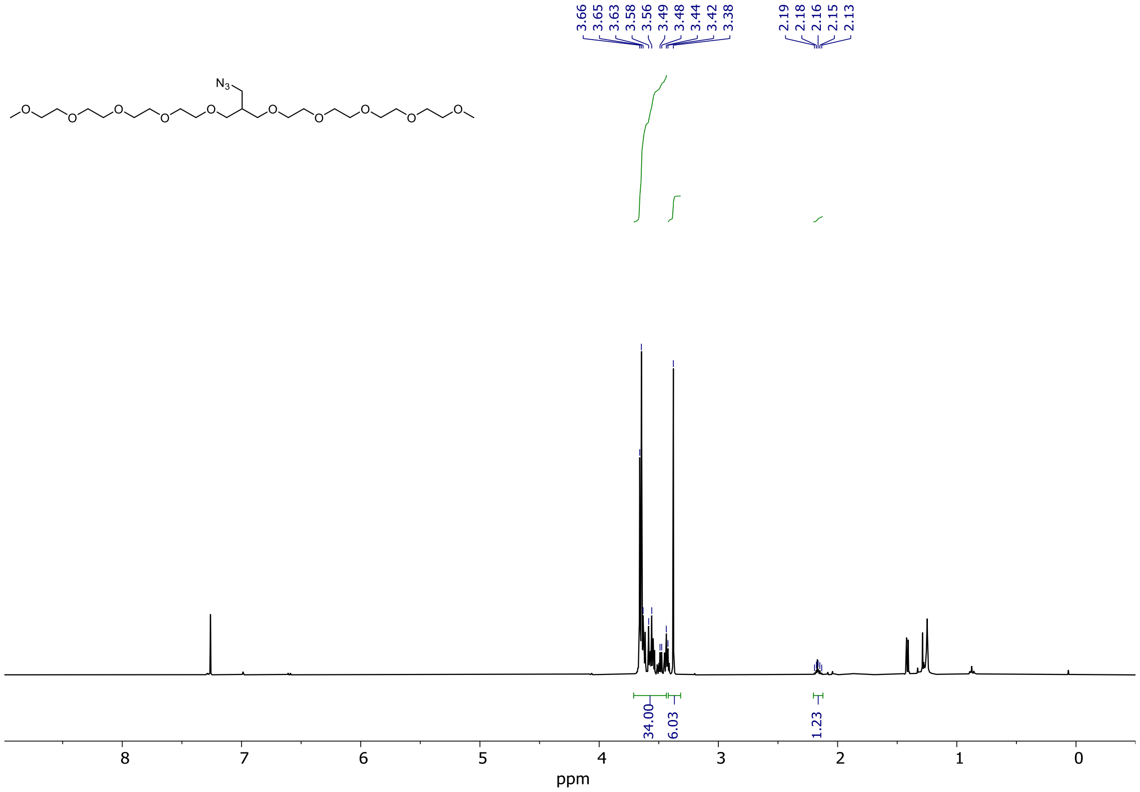

Figure S21. ^{1}H NMR spectra of **S5** in chloroform-*d*.

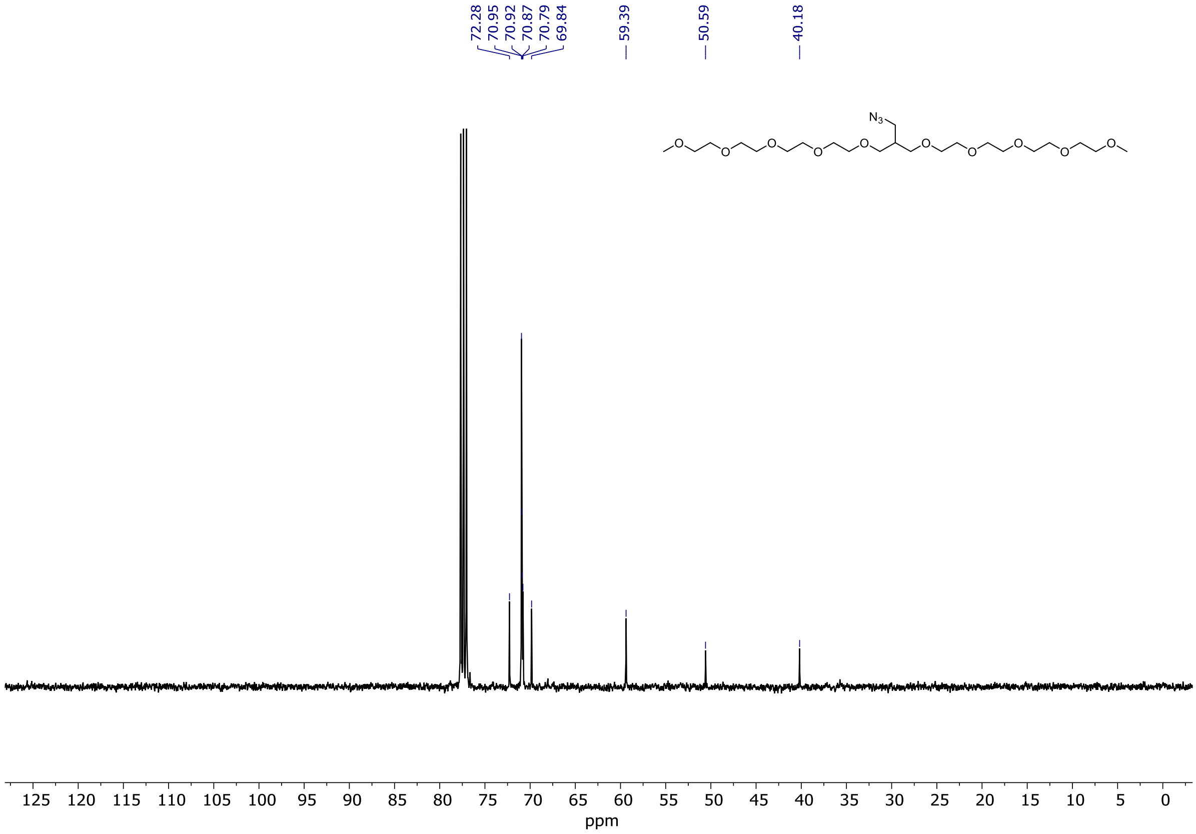

Figure S22. ^{13}C NMR spectra of **S5** in chloroform-*d*.

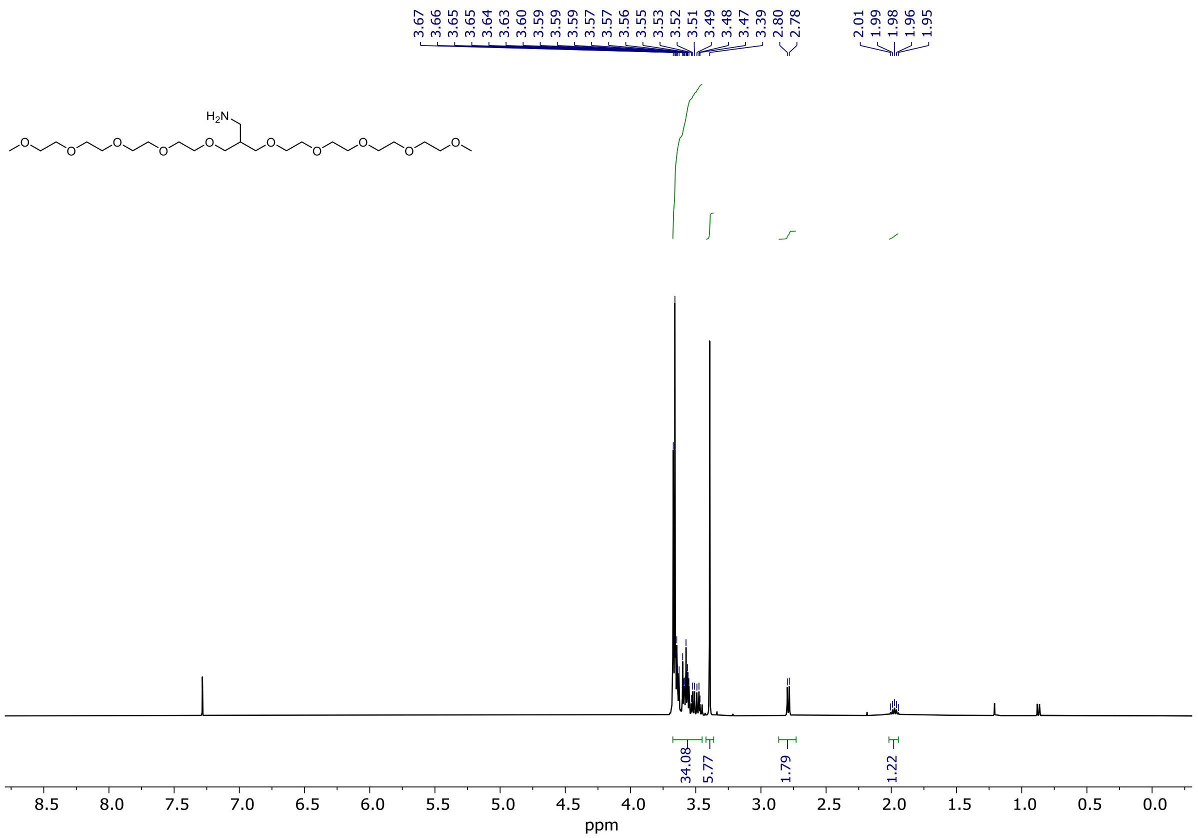

Figure S23. ^{1}H NMR spectra of **S6** in chloroform-*d*.

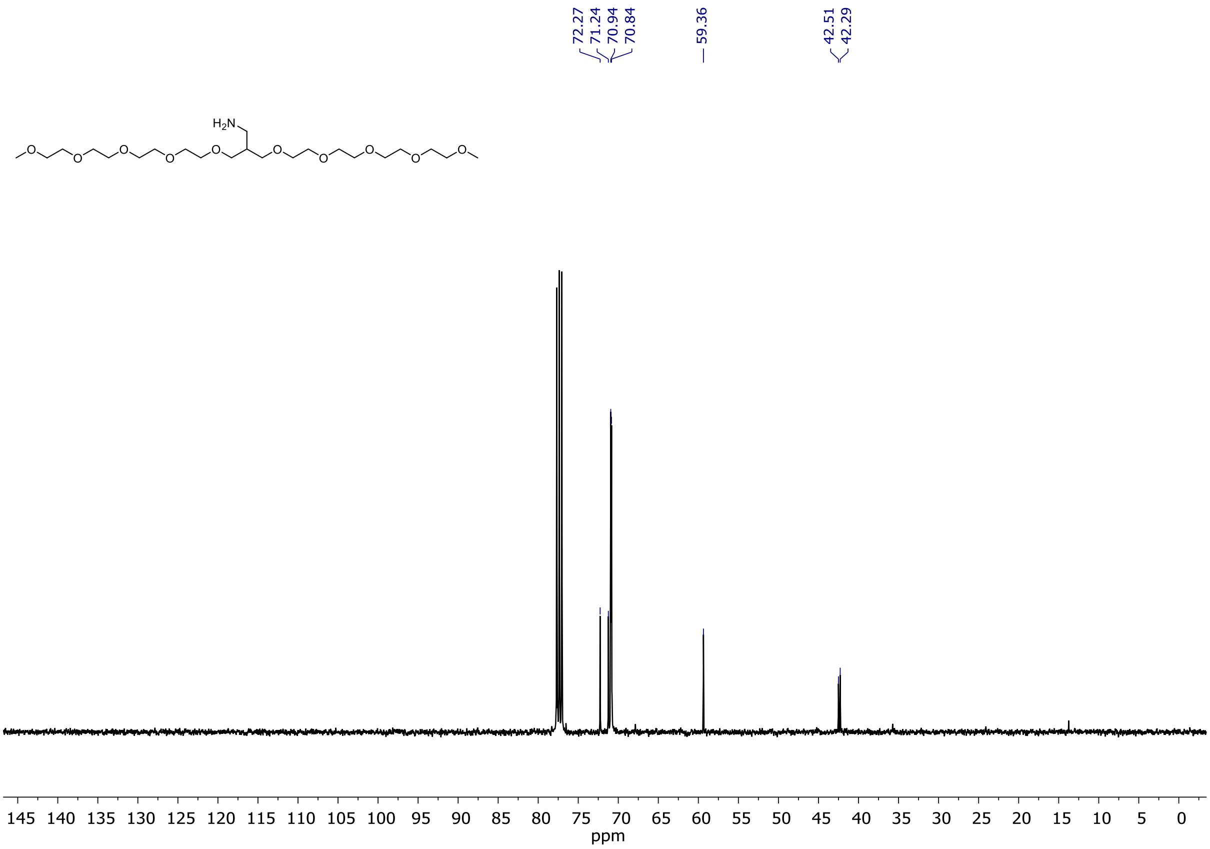

Figure S24. ^{13}C NMR spectra of **S6** in chloroform-*d*.

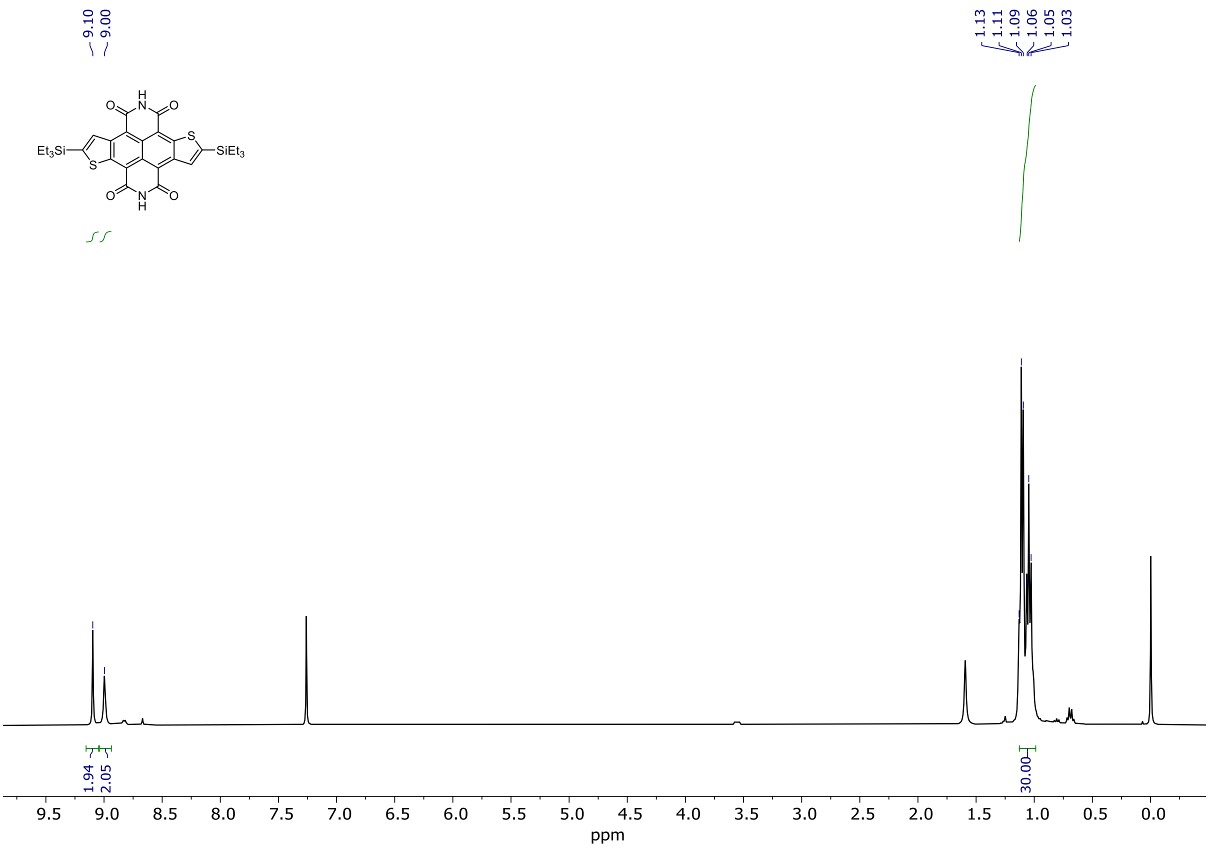

Figure S25. ^{1}H NMR spectra of **4** in chloroform-*d*.

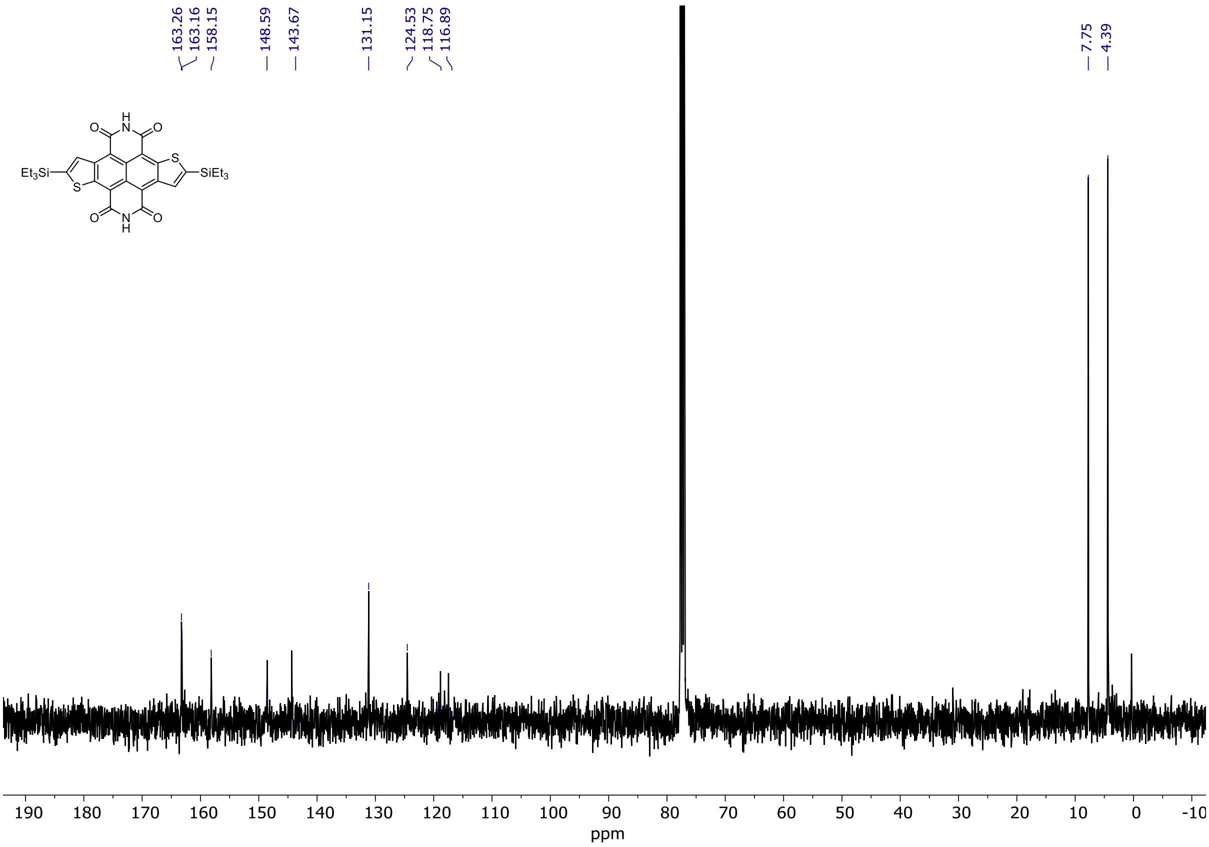

Figure S26. ^{13}C NMR spectra of **4** in chloroform-*d*.

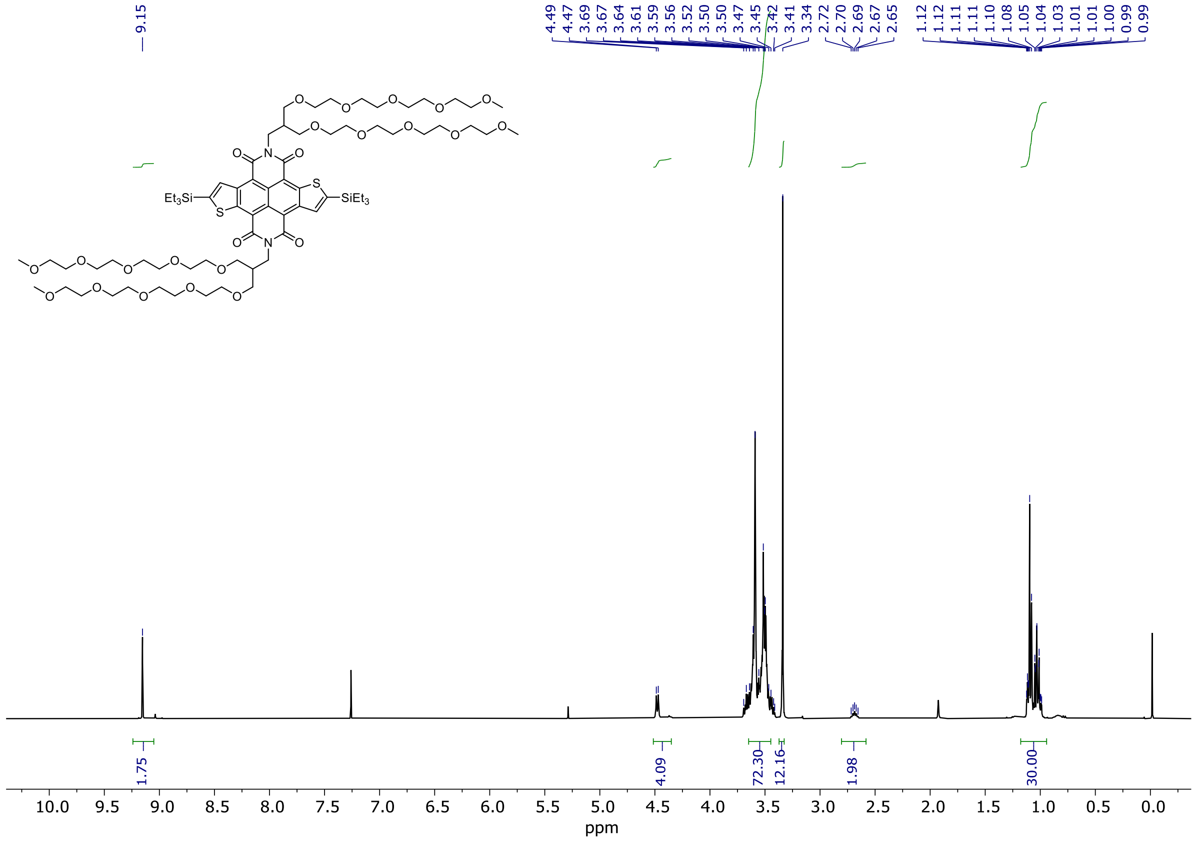

Figure S27. ^{1}H NMR spectra of **4gNDTI-TES** in chloroform-*d.*

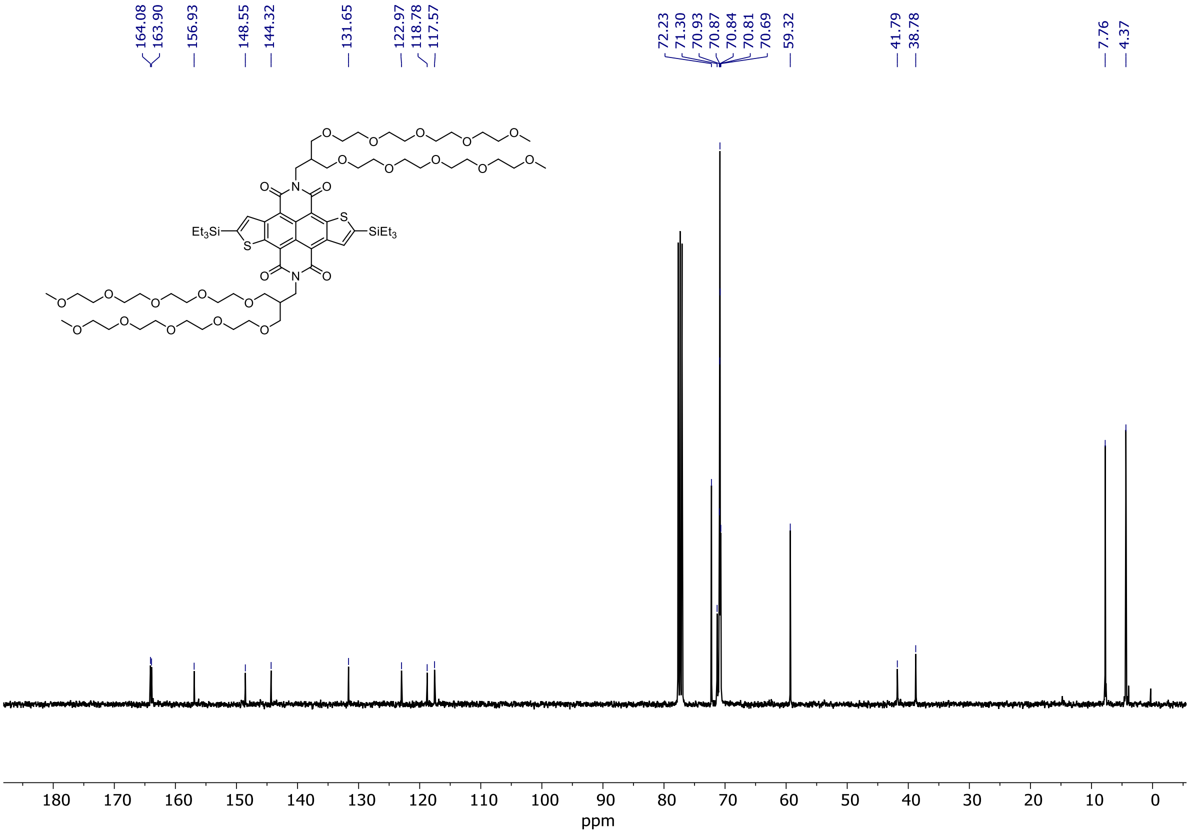

Figure S28. ^{13}C NMR spectra of **4gNDTI-TES** in chloroform-*d.*

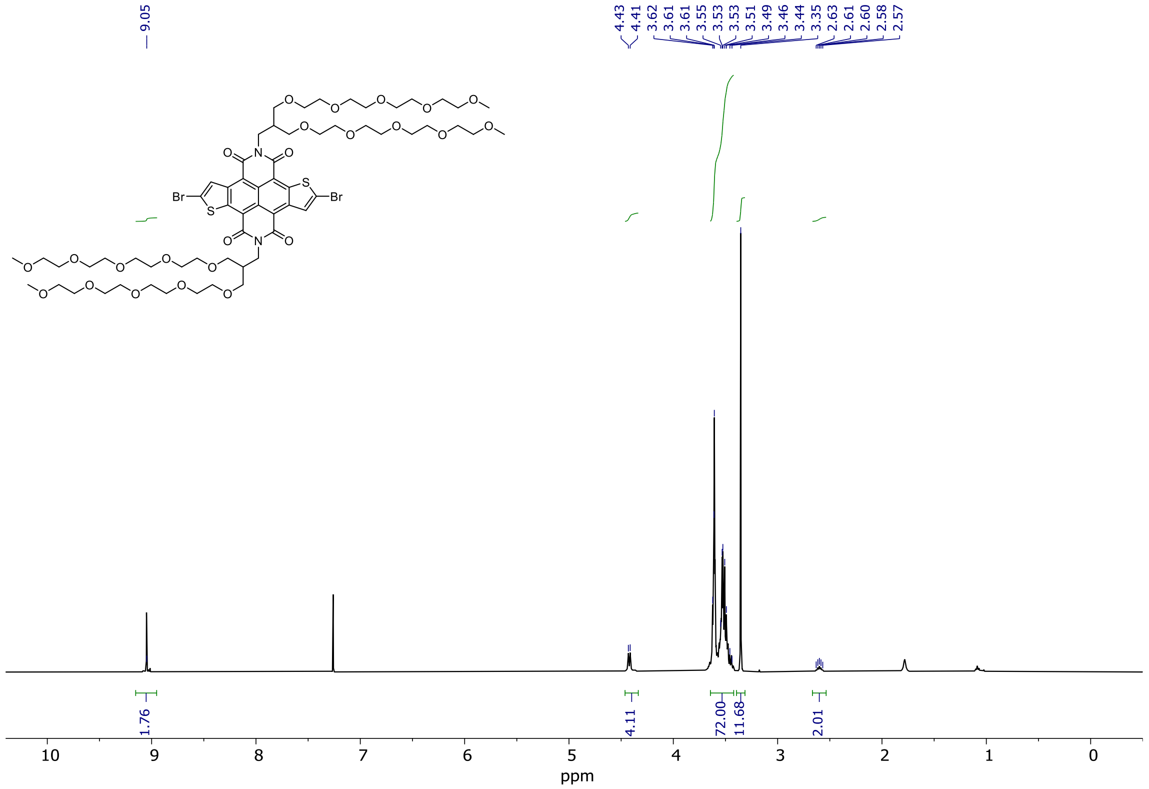

Figure S29. ^{1}H NMR spectra of **4gNDTI-Br** in chloroform-*d*.

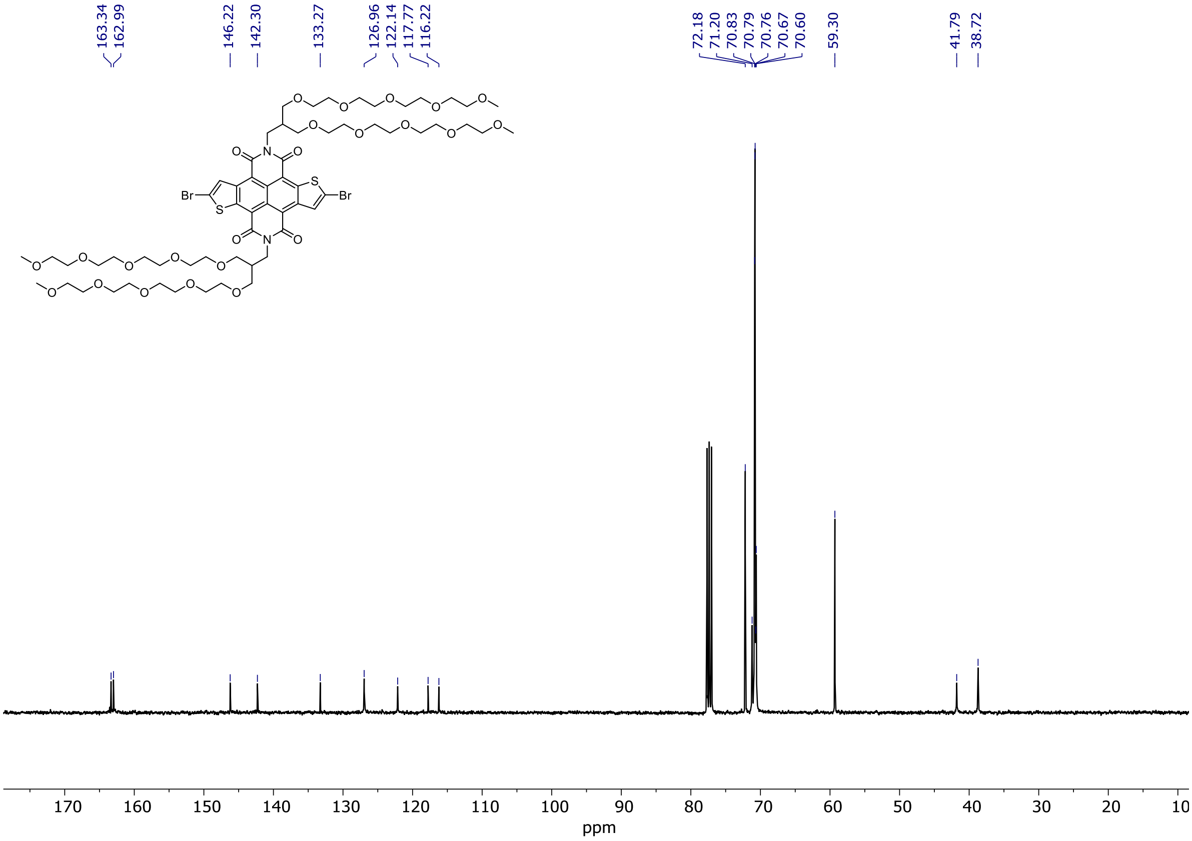

Figure S30. ^{13}C NMR spectra of **4gNDTI-Br** in chloroform-*d*.

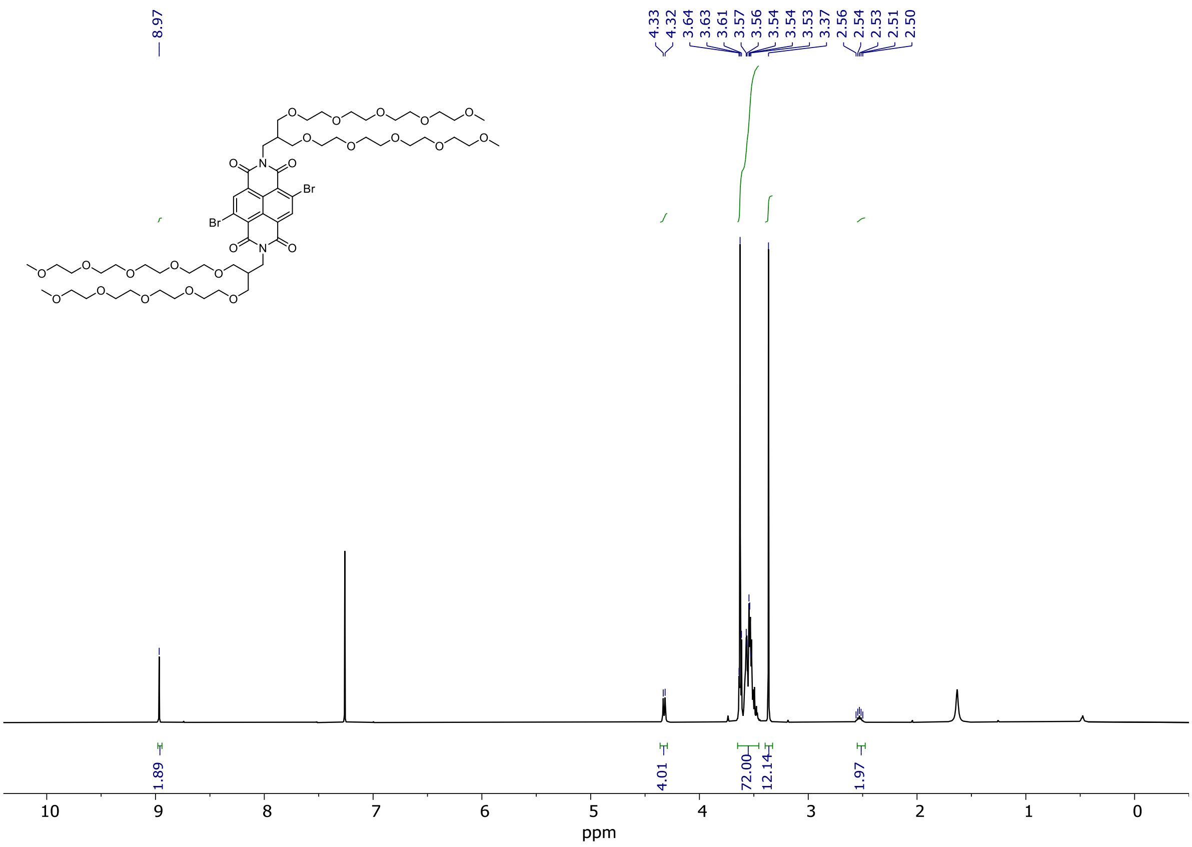

Figure S31. ^{1}H NMR spectra of **4gNDI-Br** in chloroform-*d*.

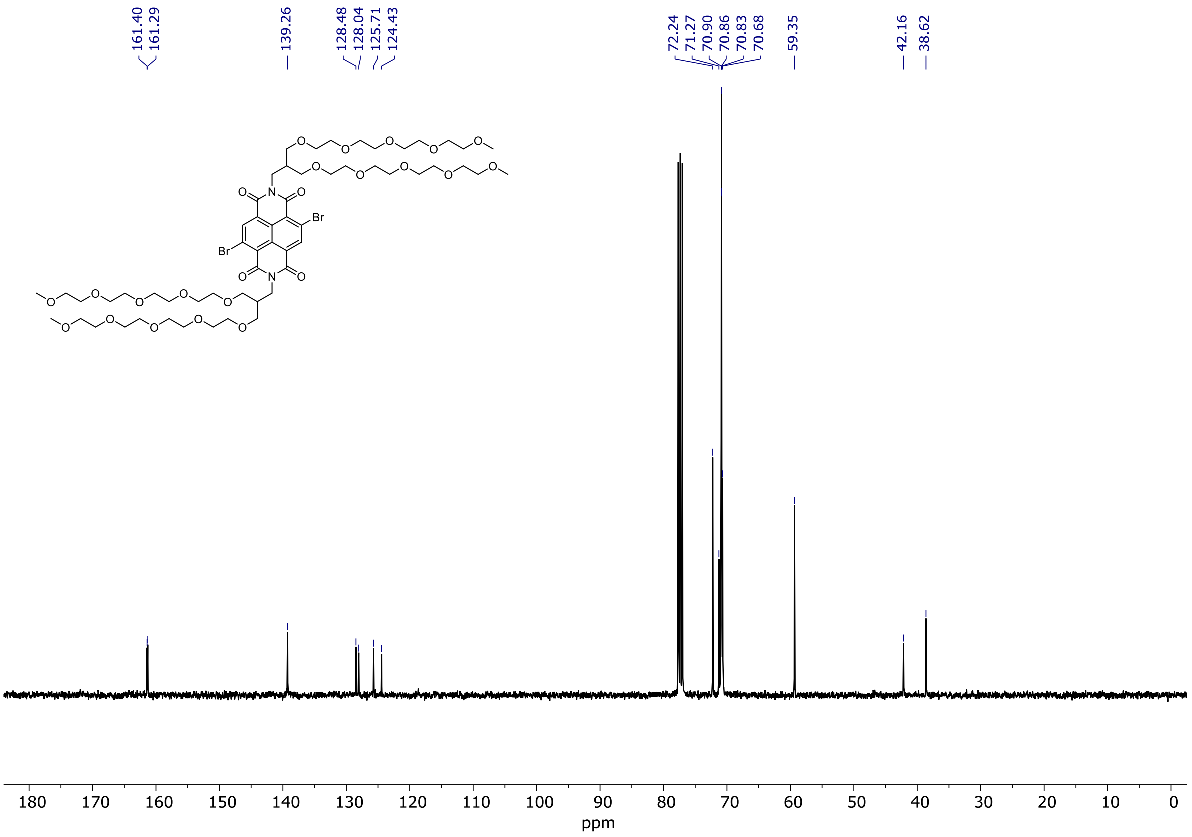

Figure S32. ^{13}C NMR spectra of **4gNDI-Br** in chloroform-*d*.

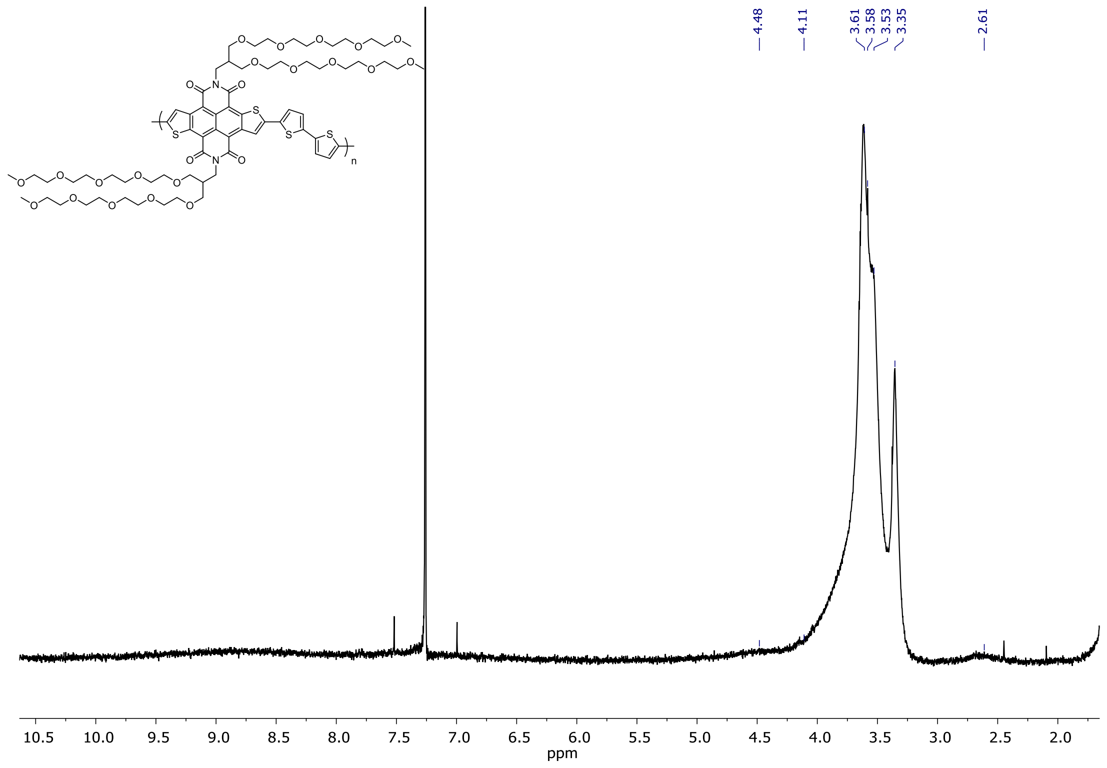

Figure S33. ^{1}H NMR spectrum of **P4gNDTI** in chloroform-*d*.

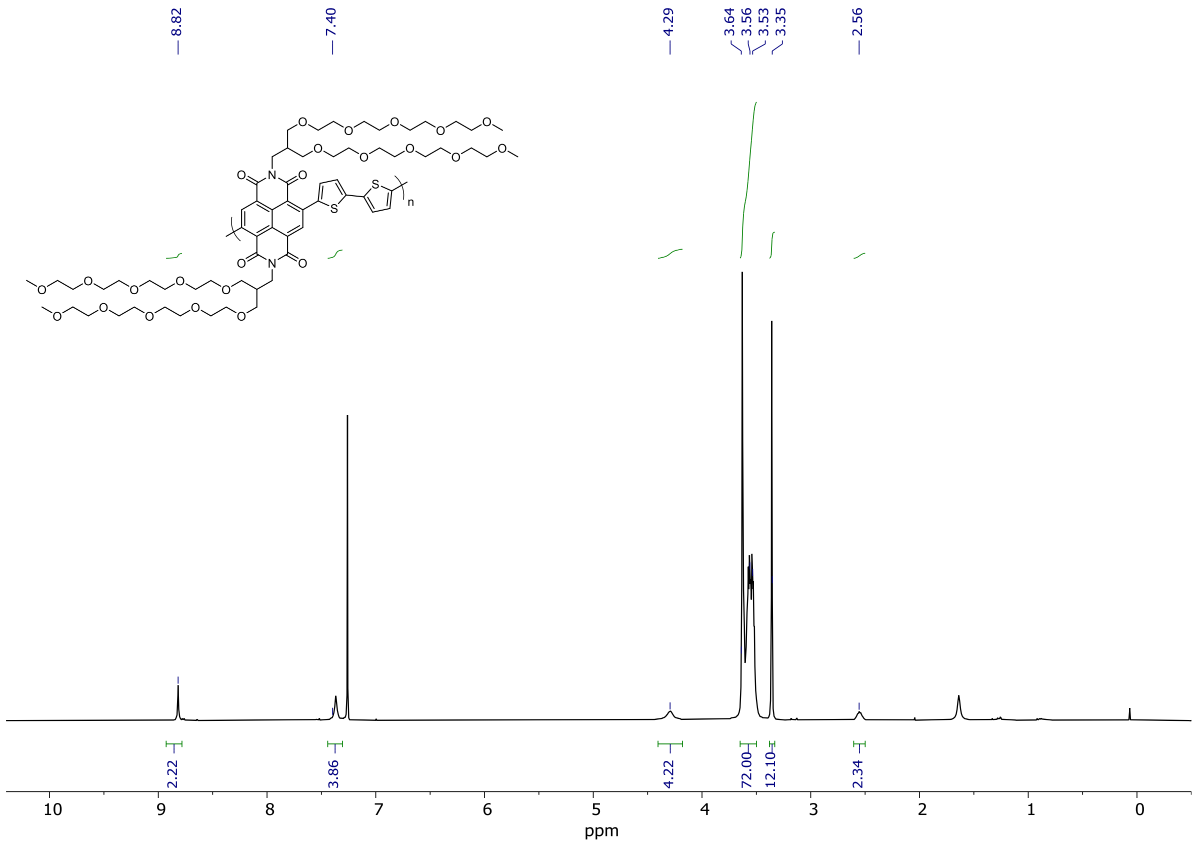

Figure S34. ^{1}H NMR spectra of **P4gNDI** in chloroform-*d*.

References

(1) Dawson, R. E.; Hennig, A.; Weimann, D. P.; Emery, D.; Ravikumar, V.; Montenegro, J.; Takeuchi, T.; Gabutti, S.; Mayor, M.; Mareda, J.; Schalley, C. A.; Matile, S. Experimental Evidence for the Functional Relevance of Anion– π Interactions. *Nat. Chem.* **2010**, *2* (7), 533–538.

(2) Nakano, M.; Sawamoto, M.; Yuki, M.; Takimiya, K. N,N′-Unsubstituted Naphthodithiophene Diimide: Synthesis and Derivatization via N-Alkylation and -Arylation. *Org. Lett.* **2016**, *18* (15), 3770–3773.
